# Modular pathway rewiring of *Saccharomyces cerevisiae* enables high-level production of L-ornithine

**DOI:** 10.1038/ncomms9224

**Published:** 2015-09-08

**Authors:** Jiufu Qin, Yongjin J. Zhou, Anastasia Krivoruchko, Mingtao Huang, Lifang Liu, Sakda Khoomrung, Verena Siewers, Bo Jiang, Jens Nielsen

**Affiliations:** 1 State Key Laboratory of Food Science and Technology, Jiangnan University, Wuxi, Jiangsu 214122, China; 2 Department of Biology and Biological Engineering, Chalmers University of Technology, Gothenburg SE-412 96, Sweden; 3 Novo Nordisk Foundation Center for Biosustainability, Chalmers University of Technology, Gothenburg SE-412 96, Sweden; 4 Novo Nordisk Foundation Center for Biosustainability, Technical University of Denmark, Hørsholm DK-2970, Denmark

## Abstract

Baker's yeast *Saccharomyces cerevisiae* is an attractive cell factory for production of chemicals and biofuels. Many different products have been produced in this cell factory by reconstruction of heterologous biosynthetic pathways; however, endogenous metabolism by itself involves many metabolites of industrial interest, and de-regulation of endogenous pathways to ensure efficient carbon channelling to such metabolites is therefore of high interest. Furthermore, many of these may serve as precursors for the biosynthesis of complex natural products, and hence strains overproducing certain pathway intermediates can serve as platform cell factories for production of such products. Here we implement a modular pathway rewiring (MPR) strategy and demonstrate its use for pathway optimization resulting in high-level production of L-ornithine, an intermediate of L-arginine biosynthesis and a precursor metabolite for a range of different natural products. The MPR strategy involves rewiring of the urea cycle, subcellular trafficking engineering and pathway re-localization, and improving precursor supply either through attenuation of the Crabtree effect or through the use of controlled fed-batch fermentations, leading to an L-ornithine titre of 1,041±47 mg l^−1^ with a yield of 67 mg (g glucose)^−1^ in shake-flask cultures and a titre of 5.1 g l^−1^ in fed-batch cultivations. Our study represents the first comprehensive study on overproducing an amino-acid intermediate in yeast, and our results demonstrate the potential to use yeast more extensively for low-cost production of many high-value amino-acid-derived chemicals.

## Abstract (web summary)

The complexity of yeast amino acid metabolism has limited carbon channelling to produce valuable chemical metabolites. Here, the authors implement a yeast customized pathway optimization strategy and demonstrate its use for overproduction of L-ornithine, an intermediate of L-arginine biosynthesis.

## Graphical abstract

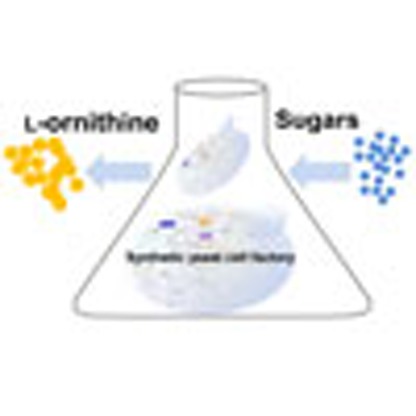

The desire to reduce our dependency on petroleum as well as increasing environmental concerns have stimulated increased efforts to develop alternative, sustainable processes for production of fuels and chemicals from renewable carbohydrate feedstocks^1^,^2^,^3^. There is also much interest in using microorganisms as cell factories for the biosynthesis of natural products possessing a wide range of biological activities^4^,^5^,^6^,^7^. Metabolic engineering offers the ability to improve biocatalysts' properties such as precision and specificity by redirecting carbon fluxes in cell factories, and in this context the concept of platform cell factories is attractive as they can be used as starting point for efficient production of a range of different chemical compounds^1^,^8^,^9^. Among the many different chemicals produced in the framework of metabolic engineering, amino-acid-derived chemicals have gained much interest^7^,^10^,^11^,^12^. Although *Escherichia coli* and *Corynebacterium glutamicum* have been intensively engineered for production of amino acids^13^ and amino-acid-derived chemicals^14^, baker's yeast *Saccharomyces cerevisiae* is an attractive host for industrial-scale processes because of its robustness and tolerance towards harsh fermentation conditions and its approval for production of many food-grade products^1^. Furthermore, several unique features of *S. cerevisiae*, including GRAS (generally recognized as safe) status, excellent availability of molecular biology tools^15^,^16^,^17^,^18^ and ability to efficiently express complex enzymes such as cytochrome P450-containing enzymes^5^,^6^,^19^, make it an even more attractive host for production of amino-acid-derived products^1^,^7^. For instance, amino-acid metabolism of this yeast has been tailored for production of higher alcohols that can serve as gasoline substitutes^11^,^20^. In addition, the pathways of aromatic amino acids including L-tryptophan, L-tyrosine and L-phenylalanine are widely used for production of various compounds, including polymer-building blocks^21^,^22^ and bioactive natural products such as benzylisoquinoline alkaloids^23^. However, the productivity and yield of these aforementioned amino-acid-derived chemicals are still very low^22^, which might be attributed to the complexity of yeast amino-acid metabolism^24^. Thus, approaches to rewire the endogenous amino-acid metabolism are required for increasing the productivity of amino-acid intermediates and low-cost production of many high-value amino-acid-derived chemicals by this cell factory.

L-ornithine, an intermediate of L-arginine biosynthesis, is widely used as a dietary supplement, as it is known to be beneficial for the treatment of wound healing and liver disease^25^. It can also be used as a precursor for production of putrescine, a diamine that is used as a nylon monomer^14^ and natural products such as tropane alkaloids, which are used as parasympatholytics for competitively antagonizing acetylcholine^26^. The biosynthesis of tropane alkaloids from L-ornithine involves P450 enzymes that are difficult to express in bacteria, and this limits the potential of bacterial systems to produce L-ornithine-derived chemicals even though L-ornithine high-producing bacterial systems have been achieved^27^,^28^,^29^. Therefore, there is much interest in developing a yeast platform cell factory for overproduction of L-ornithine.

Despite extraordinary results of amino-acid production, including L-ornithine, with *C. glutamicum*^28^,^29^,^30^, the complexity of yeast amino-acid metabolism still calls for different engineering strategies. Indeed, L-ornithine metabolism is notable for its complexity in terms of regulation and its connection with several other pathways, such as biosynthesis of polyamine and pyrimidine, and the urea cycle. L-arginine biosynthesis is compartmentalized with the synthesis of L-ornithine in the mitochondria, but using L-glutamate as substrate, which is produced in the cytoplasm^31^. After being transported to the cytoplasm, L-ornithine is converted to L-arginine in three consecutive steps. In addition, α-ketoglutarate, the precursor of L-glutamate, is one of the intermediates of the TCA cycle, the flux of which is limited in the presence of high glucose concentrations due to the so-called Crabtree effect^32^. From this it is obvious that engineering *S. cerevisiae* to overproduce L-ornithine is a daunting task.

According to the above-mentioned characteristics of L-ornithine metabolism, there are at least four challenges in constructing an L-ornithine-overproducing yeast strain. First, how to tune the metabolic flux to produce L-ornithine and not convert this further into L-arginine without incapacitating cell growth. Second, how to balance and coordinate the corresponding pathways and enzymes in different subcellular organelles, as pathway perturbation could create a limitation in the transport of intermediates resulting in the redirection of these towards competing pathways. Third, how to increase the supply of the precursor α-ketoglutarate, as the Crabtree effect will limit the TCA cycle efficiency for α-ketoglutarate biosynthesis during normal batch fermentations with excess glucose. Last, but not least, overall pathway optimization calls for intensive perturbations of interacting pathways; thus, fast and facile strategies should be implemented to achieve total pathway optimization.

To overcome these challenges, here we re-cast the L-ornithine biosynthesis of yeast into three modules on the basis of the pathway architecture, using a modular pathway rewiring (MPR) strategy, and we then completely rewire the L-arginine metabolism to drive increased metabolic flux towards L-ornithine. For each strategy we evaluate multiple metabolic engineering targets resulting in evaluation of more than 37 different genetic modifications. Through combination of many of the different metabolic engineering strategies we, in total, construct and evaluate more than 64 engineered yeast strains, and hereby gain much new insight into the regulation of L-arginine biosynthesis in yeast. The best-performing strains have significant rewiring of its arginine metabolism resulting in L-ornithine production with a titre of 1,041±47 mg l^−1^ in shake-flask experiments and 5.1 g l^−1^ in fed-batch fermentations. Our successful proof-of-concept production of L-ornithine in *S. cerevisiae* represents the first systematic case study to produce amino acids in yeast, and it demonstrates the potential to use yeast as a cell factory platform for the low-cost production of amino-acid-derived chemicals.

## Results

### Re-casting L-ornithine biosynthesis into three modules

**Figure 1:**
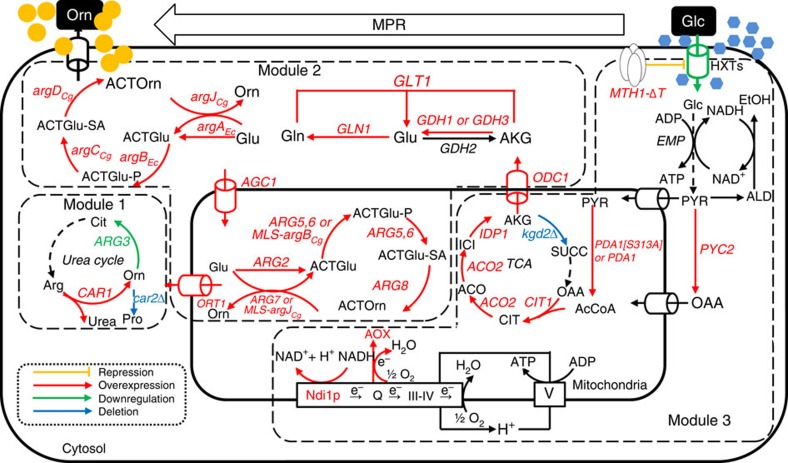
Schematic overview of L-ornithine biosynthesis in *S. cerevisiae* and the main metabolic engineering targets implemented in this study. Genes or proteins subject to overexpression are shown in red font, while those subject to downregulation or deletion are shown in green and blue, respectively. Solid arrows represent single reaction steps, while the dashed arrows represent multiple reaction steps. The green arrows represent the related genes or proteins subject to downregulation, while the red arrows indicate that the related genes or proteins are subject to overexpression. The blue arrows indicate that the related genes are subject to deletion. *CAR1*, arginase; *CAR2*, L-ornithine transaminase; *ARG3*, ornithine carbamoyltransferase; *ARG2*, glutamate N-acetyltransferase; *ARG5,6*, acetylglutamate kinase and N-acetyl-gamma-glutamyl-phosphate reductase; *ARG7*, mitochondrial ornithine acetyltransferase; *ARG8*, acetylornithine aminotransferase; *MLS-argJ*_*Cg*_, ornithine acetyltransferase from *C. glutamicum* located to the mitochondria of *S. cerevisiae*; *MLS-argB*_*Cg*_, acetylglutamate kinase from *C. glutamicum* located to the mitochondria of *S. cerevisiae*; *argA*_*Ec*_, glutamate N-acetyltransferase from *E. coli*; *argB*_*Ec*_, acetylglutamate kinase from *E. coli*; *argC*_*Cg*_, N-acetyl-gamma-glutamyl-phosphate reductase from *C. glutamicum*; *argD*_*Cg*_, acetylornithine aminotransferase from *C. glutamicum; argJ*_*Cg*_, ornithine acetyltransferase from *C. glutamicum*; *ORT1*, ornithine transporter of the mitochondrial inner membrane; *AGC1*, glutamate uniporter; *ODC1*, transporter of α-ketodicarboxylate or α-ketoglutarate of the mitochondrial inner membrane; *GDH1*, NADP^+^-dependent glutamate dehydrogenase; *GDH3*, NADP^+^-dependent glutamate dehydrogenase; *GLT1*, NAD^+^-dependent glutamate synthase; *GLN1*, glutamine synthetase; *GDH2*, NAD^+^-dependent glutamate dehydrogenase; *CIT1*, citrate synthase; *PYC2*, pyruvate carboxylase isoform; *ACO2*, putative mitochondrial aconitase isozyme; *IDP1*, mitochondrial NADP^+^-specific isocitrate dehydrogenase; *PDA1*, E1 alpha subunit of the pyruvate dehydrogenase (PDH) complex; *PDA1[S313A]*, *PDA1* with mutation S313A; *KGD2*, dihydrolipoyl transsuccinylase; *MTH1-ΔT*, truncated version of *MTH1*; Ndi1p, NADH:ubiquinone oxidoreductase. TCA, tricarboxylic acid cycle; EMP, the glycolysis pathway; HXT, hexose transporter; AOX, NADH alternative oxidase; Q, ubiquinone. Respiratory chain (complexes III–IV) in the mitochondrial inner membrane is shown as a rectangle, while the ATP synthase (complex V) is shown as a square. Orn, L-ornithine; Glu, L-glutamate; Gln, L-glutamine; Arg, L-arginine; Cit, L-citrulline; Pro, L-proline; ACTGlu, N-acetyl-L-glutamate; ACTGlu-P, N-acetylglutamyl-P; ACTGlu-SA, N-acetylglutamate semialdehyde; ACTOrn, N-acetylornithine; AKG, α-ketoglutarate; CIT, citrate; ACO, aconitate; ICI, isocitrate; OAA, oxaloacetate; AcCoA, acetyl CoA; PYR, pyruvate; SUCC, succinyl-CoA; EtOH, ethanol; ALD, acetaldehyde; Glc, glucose.

Overall optimization of L-ornithine biosynthesis calls for perturbations of a large number of genes (Fig. 1 and Supplementary Fig. 1), and we therefore used a MPR strategy that was based on DNA assembly^18^ and modular pathway engineering (MOPE) methods^4^ that enabled efficient optimization of the overall pathway from glucose to L-ornithine. Hereby, the overall pathway from glucose to L-ornithine was re-cast into three modules as follows. Module 1: L-ornithine degradation or consumption module. When L-ornithine, which is synthesized in the mitochondria, is transported to the cytoplasm, subsequent reactions can transform it to L-citrulline or L-glutamate γ-semialdehyde by ornithine carbamoyltransferase (OTC; *ARG3*) and ornithine aminotransferase (*CAR2*), respectively. The arginase reaction (*CAR1*) that degrades L-arginine into urea and L-ornithine was also included in this module (Supplementary Fig. 2); Module 2: L-ornithine synthesis module. This module contains all the reactions by which the TCA cycle intermediate α-ketoglutarate is converted to L-ornithine. Following L-glutamate transport into the mitochondria from the cytoplasm, five reaction steps convert it to L-ornithine. This cyclic L-ornithine synthesis pathway is the so-called acetylated derivative cycle because the acetyl group that is added to L-glutamate in the initial step is recycled from N-acetyl-L-ornithine generated in the fifth step (Supplementary Fig. 3); Module 3: α-ketoglutarate synthesis module. This module includes glucose uptake reactions (including its regulation), glycolysis and the upstream part of the TCA cycle. The respiratory chain, which is highly associated with the TCA cycle flux, is also included in this module (Supplementary Fig. 4).

Our MPR approach was initiated with engineering of the downstream Module 1 of L-ornithine consumption, and then the strain with the optimized downstream module was subject to upstream module optimization. Throughout the manuscript, strain names indicate the modules present in the strain^33^, for example, M1aM2bM3c includes strategy a for Module 1, strategy b for Module 2 and strategy c for Module 3. See Table 1 and Supplementary Tables 1 and 2 for descriptions of all strains constructed in connection with pathway optimization.

### Leaky arginine auxotrophy enables L-ornithine production

**Figure 2:**
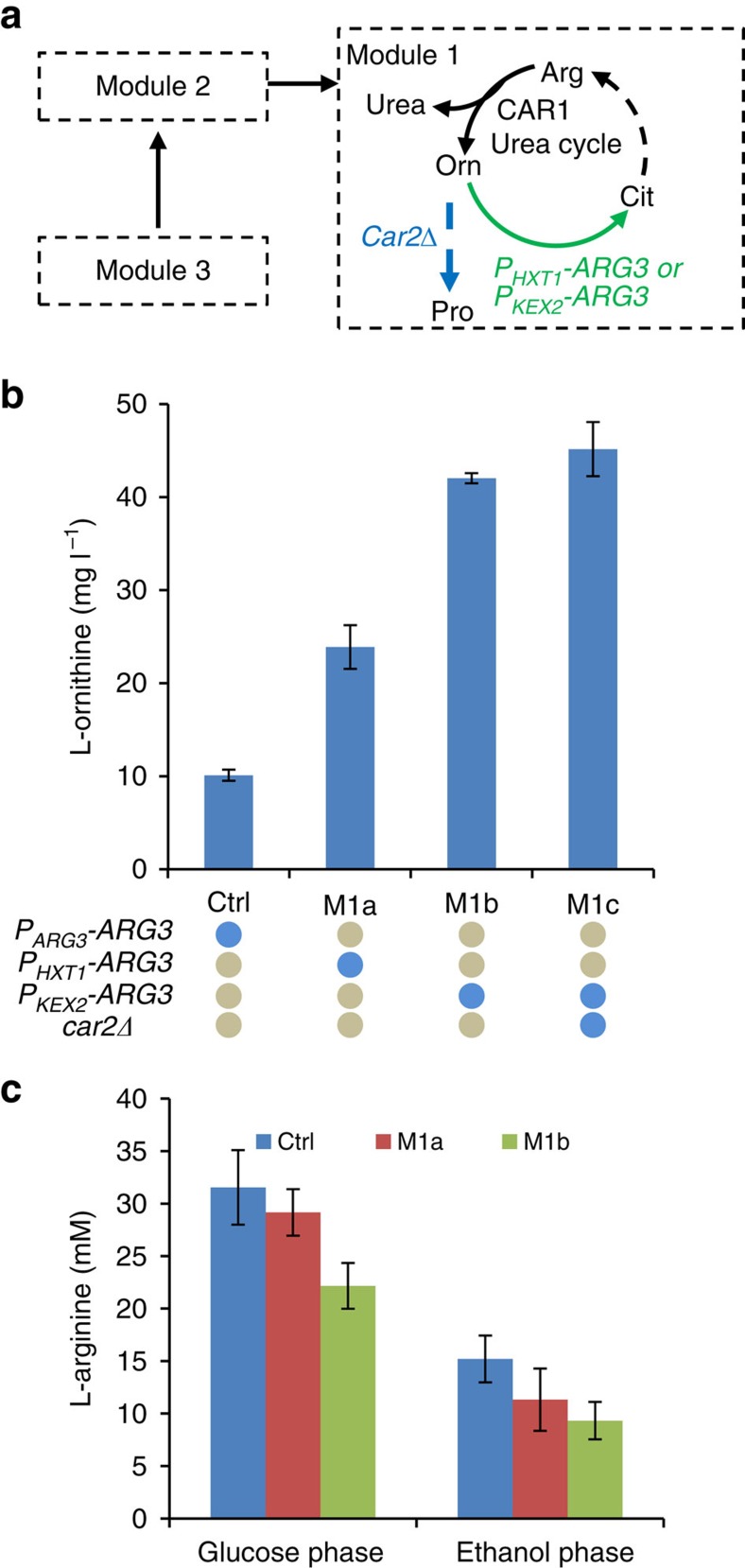
Leaky L-arginine auxotrophy enables L-ornithine overproduction. (**a**) The MPR was initiated from Module 1. *CAR1*, arginase; *CAR2*, L-ornithine transaminase; *ARG3*, ornithine carbamoyltransferase. See Figure 1 legend regarding abbreviations. The attenuation of *ARG3* was implemented by replacing the original promoter of *ARG3* with the *HXT1* or *KEX2* promoter (*P*_*HXT1*_*-ARG3* and *P*_*KEX2*_*-ARG3*, respectively). (**b**) The transcriptional downregulation of Arg3p and knockout of L-ornithine potential consumption step Car2p led to L-ornithine overproduction. Blue solid circle indicates that the molecular implementation is included in the strain under test. Cells were grown in defined minimal medium with 20 gl^−1^ glucose and cultures were sampled after 72 h of growth for L-ornithine detection. Displayed are the average values±s.d. from at least three biological replicates. (**c**) The transcriptional downregulation of Arg3p decreased the intracellular L-arginine pool. Cells were grown in defined minimal medium with 20 gl^−1^ glucose and cultures were sampled in both glucose phase (OD_600_ is between 0.8 and 1.2 approximately) and ethanol phase (OD_600_ is up to 4–5) for L-arginine and biomass detection. All data are presented as the mean±s.d. (*n*≥3).

For L-arginine biosynthesis, L-ornithine is converted to L-citrulline by OTC (*ARG3*)^31^ in the cytoplasm after L-ornithine is exported from the mitochondria. The biosynthesis of L-ornithine is hereby controlled by the presence of L-arginine because of feedback inhibition and repression of the key enzymes^34^. Blocking the consumption pathway of target chemicals completely is a common strategy for accumulation of desired chemicals, and this strategy has been successfully applied for L-ornithine production by *C. glutamicum*, where the OTC-encoding genes were fully deleted^27^,^35^. However, the entailed L-arginine auxotrophy of the resulting strains requires L-arginine supplementation, which might add costs and cause problems for large-scale operation. We therefore fine-tuned *ARG3* expression rather than deleting the gene so that L-arginine can still be synthesized at low levels to support growth, and this will also limit any negative regulation of L-arginine on L-ornithine biosynthesis. We weakened *ARG3* expression by replacing its native promoter with either the glucose-regulated *HXT1* promoter (*HXT1* encodes a low-affinity glucose transporter of the major facilitator superfamily) or the low-activity *KEX2* promoter (*KEX2* encodes a protein-processing Ca^2+^-dependent serine protease)^36^,^37^ (Fig. 2a). Both strain M1a (*HXT1* promoter) and M1b (*KEX2* promoter) accumulated and secreted L-ornithine (Fig. 2b, Supplementary Figs 5 and 6). While strain M1a produced 24 mg l^−1^ L-ornithine, strain M1b had a 76% higher titre of 42 mg l^−1^ (Fig. 2b). It is interesting that the intracellular L-arginine level only decreased ∼30% compared with the control (Fig. 2c). It is apparent that the synthesis of L-arginine was to some extent maintained despite the low-level expression of *ARG3*. We speculated that the L-ornithine biosynthetic pathway was upregulated by the transcription factor Gcn4p (refs 24, 38) to alleviate decreased intracellular L-arginine levels following Arg3p downregulation, and this may have ensured L-arginine biosynthesis through the upregulation of genes in the L-ornithine biosynthetic pathway (Supplementary Fig. 7).

L-ornithine can also be transaminated to L-glutamate γ-semialdehyde by ornithine aminotransferase (Car2p), and L-glutamate γ-semialdehyde can be further converted into L-glutamate in the presence of oxygen. Deletion of *CAR2* may improve L-ornithine production by blocking this futile cycle. However, deletion of *CAR2* in M1b (strain M1c) resulted in only a small increase in the L-ornithine titre to 45 mg l^−1^ (Fig. 2b). This small effect could be attributed to lack of a functional transaminase reaction at the low L-ornithine level (42 mg l^−1^) in strain M1b. However, as the Car2p reaction may be activated when the whole pathway would be further improved for L-ornithine production, we thus used strain M1c harbouring the *CAR2* deletion as the parent strain for the subsequent pathway optimization.

### Subcellular trafficking engineering and pathway translocation

**Figure 3:**
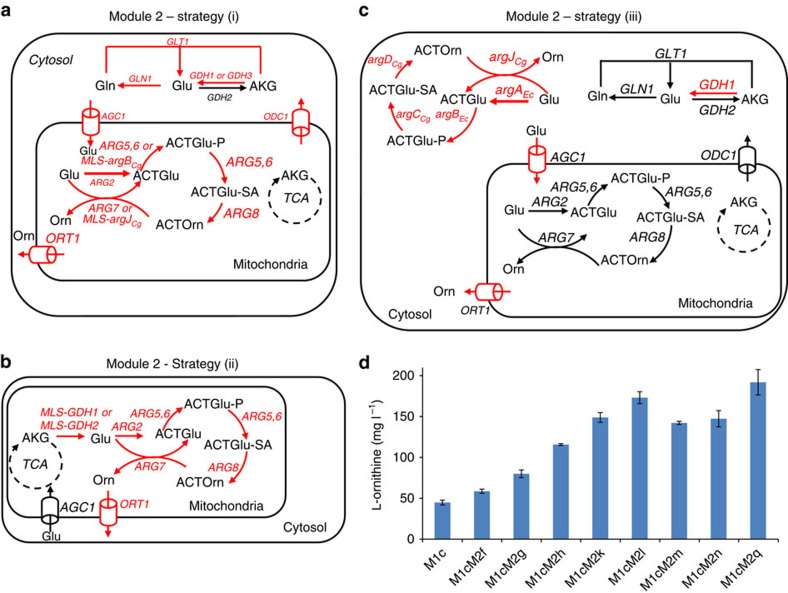
Subcellular trafficking engineering and pathway translocation elevates L-ornithine synthesis. To coordinate transport and biosynthesis of L-ornithine, we applied three different strategies: (**a**) improving mitochondrial L-ornithine biosynthesis combined with engineering of transporters [strategy (i)]; (**b**) improving biosynthesis of mitochondrial L-ornithine biosynthesis combined with translocation of glutamate biosynthesis to the mitochondria [strategy (ii)]; and (**c**) translocation of the whole L-ornithine biosynthetic pathway to the cytoplasm [strategy (iii)]. In **a**–**c**, fonts and arrows are as described in legend to Fig. 1. *ARG2*, glutamate N-acetyltransferase; *ARG5,6*, acetylglutamate kinase and N-acetyl-gamma-glutamyl-phosphate reductase; *ARG7*, mitochondrial ornithine acetyltransferase; *ARG8*, acetylornithine aminotransferase; *MLS-argJ*_*Cg*_, ornithine acetyltransferase from *C. glutamicum* located to the mitochondria of *S. cerevisiae*; *MLS-argB*_*Cg*_, acetylglutamate kinase from *C. glutamicum* located to the mitochondria of *S. cerevisiae*; *argA*_*Ec*_, glutamate N-acetyltransferase from *E. coli*; *argB*_*Ec*_, acetylglutamate kinase from *E. coli*; *argC*_*Cg*_, N-acetyl-gamma-glutamyl-phosphate reductase from *C. glutamicum*; *argD*_*Cg*_, acetylornithine aminotransferase from *C. glutamicum; argJ*_*Cg*_, ornithine acetyltransferase from *C. glutamicum*; *ORT1*, ornithine transporter of the mitochondrial inner membrane; *AGC1*, glutamate uniporter; *ODC1*, transporter of α-ketodicarboxylate or α-ketoglutarate of the mitochondrial inner membrane; *GDH1*, NADP^+^-dependent glutamate dehydrogenase; *GDH3*, NADP^+^-dependent glutamate dehydrogenase; *GLT1*, NAD^+^-dependent glutamate synthase; *GLN1*, glutamine synthetase; *GDH2*, NAD^+^-dependent glutamate dehydrogenase; *MLS-GDH1*, mitochondrially targeted NADP^+^-dependent glutamate dehydrogenase; *MLS-GDH2*, mitochondrially targeted NAD^+^-dependent glutamate dehydrogenase. See Fig. 1 legend regarding abbreviations of metabolites. (**d**) Pathway variants in Module 2 enable substantial increase in L-ornithine titre. Cells were grown in defined minimal medium with 20 gl^−1^ glucose, and cultures were sampled after 72 h of growth for L-ornithine detection. Displayed is the average values±s.d. (*n*≥3).

After optimization of L-ornithine consumption, we set out to optimize Module 2 of the L-ornithine biosynthesis pathway from α-ketoglutarate. This part of L-ornithine biosynthesis is notable for its compartmentalization, where the key metabolites, such as L-glutamate, α-ketoglutarate and L-ornithine, are synthesized in different compartments. Thus, engineering of the specific biosynthetic pathway should be coordinated with the transport of these intermediates. To coordinate transport and biosynthesis of L-ornithine, we applied three different strategies. First, improving mitochondrial L-ornithine biosynthesis combined with engineering of transporters (Fig. 3a). Second, improving mitochondrial L-ornithine biosynthesis combined with translocation of L-glutamate biosynthesis to the mitochondria (Fig. 3b). Last, but not least, translocation of the entire L-ornithine biosynthetic pathway to the cytoplasm (Fig. 3c).

The first two strategies involved overexpressing genes of the mitochondrial L-ornithine biosynthetic pathway from L-glutamate. As expected, overexpression of *ARG5,6*, *ARG7* and *ARG8* in strain M1cM2f resulted in an increased L-ornithine titre of 59 mg l^−1^, representing a 31% increase compared with the control strain M1c (Fig. 3d and Supplementary Fig. 8). We also evaluated overexpression of each of the individual steps of the pathway to identify if a single enzyme should be flux-controlling, but single enzyme overexpression did not result in any improvement in titre over the M1c strain (Supplementary Fig. 9). This part of the pathway is the so-called acetylated derivative cycle, where there is cycling of the acetyl group. We speculated that if the level of acetylated intermediates is increased there will be a higher catalytic activity of the pathway, and we therefore also overexpressed N-acetylornithine synthase encoded by *ARG2* resulting in strain M1cM2g producing 80 mg l^−1^ of L-ornithine, a 36% increase compared with parent strain M1cM2f (Fig. 3d and Supplementary Fig. 8).

We also evaluated overexpression of the positive regulator transcription factor Gcn4p (refs 24, 38) as an alternative strategy for overexpression of *ARG5,6*, *ARG7* and *ARG8* in the L-ornithine biosynthetic pathway. We used promoters of different strengths^38^ to fine-tune expression of a truncated version of *GCN4* (*tGCN4*), in which the residues 99–106 were truncated to circumvent rapid degradation of Gcn4p through the ubiquitin pathway^38^. However, there was no obvious difference in L-ornithine titre (Supplementary Fig. 10).

L-ornithine is exported from the mitochondria to the cytosol by Ort1p (ref. 39) where it is used for L-arginine biosynthesis. In the first strategy we therefore increased the expression level of *ORT1* and when this was performed in the M1cM2g strain, resulting in strain M1cM2h, the L-ornithine titre increased to 115 mg l^−1^, representing a 44% increase (Fig. 3d and Supplementary Fig. 8). Encouraged by these findings we also overexpressed *AGC1,* the gene encoding the glutamate uniporter/aspartate–glutamate exchanger^40^, to ensure sufficient supply of L-glutamate for L-ornithine biosynthesis. By overexpression of *AGC1* in strain M1cM2h, resulting in strain M1cM2k, L-ornithine titre increased to 149 mg l^−1^, representing a further 30% increase (Fig. 3d and Supplementary Fig. 8). Both strategies point to that internal trafficking may often be rate-limiting in metabolic pathways, and overexpressing related transporters may be an efficient strategy for boosting the pathway flux to chemicals of interest.

Finally, as part of the first strategy we engineered L-glutamate biosynthesis in the cytosol. There are three pathways for L-glutamate biosynthesis in *S. cerevisiae*. Two of these are mediated by the two isoforms of NADPH-dependent glutamate dehydrogenase, encoded by *GDH1* and *GDH3,* while the third pathway involves combined activities of glutamine synthetase (encoded by *GLN1*) and glutamate synthase (encoded by *GLT1*)^41^. We overexpressed each of the three pathways in strain M1cM2k, resulting in strains M1cM2l (*GDH1*), M1cM2m (*GDH3*) and M1cM2n (*GLN1* and *GLT1*). Of these targets, overexpression of *GDH1* (strain M1cM2l) resulted in significant improvement in L-ornithine production, that is, a 16% increase in final titre corresponding to 173 mg l^−1^ (Fig. 3d). This indicates that Gdh1p, which generally is considered the key route for L-glutamate biosynthesis at ammonia access, is the most efficient one for L-glutamate biosynthesis, probably because of its high affinity for α-ketoglutarate^42^. Subsequently, to boost the cytoplasmic α-ketoglutarate pool for L-glutamate synthesis, we also overexpressed Odc1p that is reported to be responsible for α-ketoglutamate transport from mitochondria to the cytosol in strains M1cM2l and M1cM2m, respectively; however, this did not result in any substantial L-ornithine titre improvement (Supplementary Fig. 11).

In the second strategy, we relocated glutamate biosynthesis into the mitochondria by re-localizing the most efficient glutamate dehydrogenase Gdh1p. As Gdh1p is NADPH-dependent and mitochondria have low levels of NADPH, we also evaluated the re-localization of Gdh2p, which is NADH-dependent. Contrary to our expectations, mitochondrial re-localization of Gdh1p (strain M1cM2i) or Gdh2p (M1cM2j) decreased the L-ornithine titre significantly (Supplementary Fig. 12). The failure of this strategy may be attributed to either poor functioning of the glutamate dehydrogenases in the mitochondrial environment or to metabolite levels disfavouring these reactions in mitochondria.

As the third strategy, we took a different approach and re-localized the complete L-ornithine biosynthetic pathway to the cytosol, where the precursor L-glutamate is synthesized. We introduced a synthetic cytosolic L-ornithine pathway in strain M1cM2q, where the genes encoding the first two enzymes, *argA*_*EC*_ and *argB*_*Ec*_, were from *E. coli* and the other three pathway genes, *argJ*_*Cg*_*, argC*_*Cg*_ and *argD*_*Cg*_, were from *C. glutamicum*, a very efficient producer of amino acids, including L-ornithine^43^. The successful growth complementation of an L-arginine auxotrophic *S. cerevisiae* strain with *ORT1* disruption demonstrated that the cytosolic pathway was functional and could provide the precursor L-ornithine for L-arginine biosynthesis (Supplementary Fig. 13). Indeed, the strain M1cM2q had an 11% increase in L-ornithine production as compared with strain M1cM2l, resulting in a titre of 192 mg l^−1^ in shake-flask fermentations (Fig. 3d). As indicated in Supplementary Figs 14 and 15 and Supplementary Table 8, the specific L-ornithine titre (mg L-ornithine per g biomass) follows the same trend as that of the L-ornithine titre among the recombinant strains as there was no substantial variation in terms of biomass concentration obtained with the different strains.

These results demonstrated that re-localization of the complete pathway for L-ornithine biosynthesis into the cytosol was more successful for L-ornithine production than engineering the endogenous pathway.

### Crabtree effect attenuation improves carbon flux to L-ornithine

**Figure 4:**
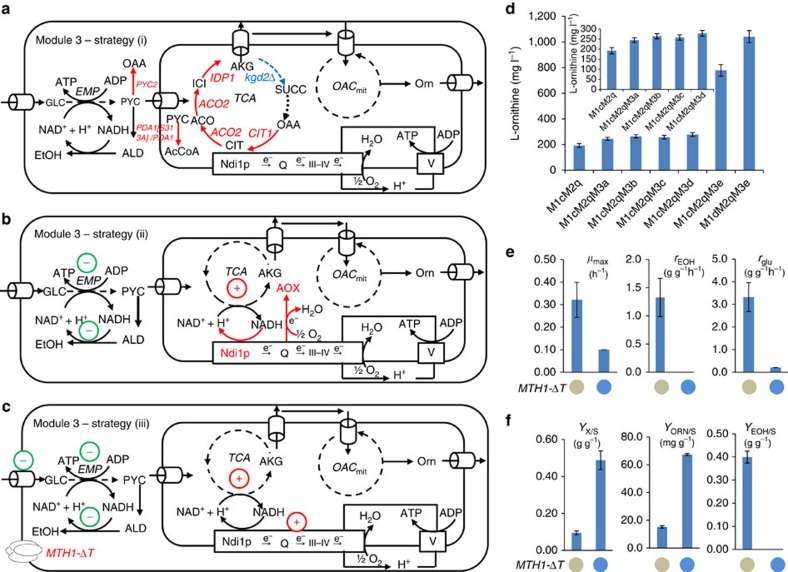
Attenuation of the ‘Crabtree effect' improves carbon channelling to L-ornithine (Module 3). To overcome the ‘Crabtree effect' we evaluated three different strategies: (**a**) strategy (i) overexpression of TCA cycle genes involved in the biosynthesis of α-ketoglutarate; (**b**) strategy (ii) improving consumption of NADH generated in connection with α-ketoglutarate biosynthesis; and (**c**) strategy (iii) attenuating the glucose uptake rate and hereby reducing overflow metabolism to ethanol. See Fig. 1 legend regarding abbreviations of genes and metabolites. (**d**) Strains with variant pathways in Module 3 led to increased production of L-ornithine. Cells were grown in defined minimal medium with 20 gl^−1^ glucose and cultures were sampled after 72 h of growth for L-ornithine quantification. Exceptionally, cultures were sampled after 108 h of growth for strain M1cM2qM3e and M1dM2qM3e as *ΔT-MTH1* overexpression impaired the glucose uptake accompanied by the decreased growth rate. Displayed is the average values±s.d. from at least three biological replicates. (**e**) Physiological characterization of the strain expressing *MTH1-ΔT* and the control. The blue solid circle represents the strain harbouring *MTH1-ΔT,* while the grey one represents the one without *MTH1-ΔT*. The specific growth rate: μ_max_ (h^−1^), specific ethanol production rate: *r*_EOH_ (g ethanol (g DCW)^−1 ^h^−1^) and specific glucose uptake rate: *r*_glu_ (g glucose (g DCW)^−1 ^h^−1^) are shown. All values were calculated in batch culture on glucose during the exponential growth phase. (**f**) Physiological characterization of the strain harbouring *MTH1-ΔT* and the control. The blue solid circle represents the strain harbouring *MTH1-ΔT,* while the grey one represents the one without *MTH1-ΔT*. The biomass yield on glucose: *Y*_*X/S*_ ((g DCW) (g glucose)^−1^), the L-ornithine yield on glucose: *Y*_ORN/S_ (mg L-ornithine (g glucose)^−1^) and the ethanol yield on glucose: *Y*_*EOH/S*_ (g ethanol (g glucose)^−1^) are shown. All values were calculated in batch cultures on glucose during the exponential growth phase. All data are presented as the mean±s.d. (*n*≥3).

After efficiently channelling α-ketoglutarate towards L-ornithine, we set out to enhance α-ketoglutarate supply by optimizing Module 3, which involves the conversion of glucose to α-ketoglutarate. However, the optimization of this part is more difficult in *S. cerevisiae* because of the Crabtree effect, that is, the majority of carbon is channelled towards ethanol via aerobic fermentation^32^ when *S. cerevisiae* is grown aerobically at high glucose concentrations. The Crabtree effect hereby compromises carbon flux to the TCA cycle that provides α-ketoglutarate required for L-ornithine biosynthesis. To overcome this problem, we evaluated three different strategies. First, overexpression of TCA cycle genes involved in the biosynthesis of α-ketoglutarate (Fig. 4a). Second, improving consumption of NADH generated in connection with α-ketoglutarate biosynthesis (Fig. 4b). Third, attenuating glucose uptake rate and hereby reducing overflow metabolism to ethanol (Fig. 4c).

Previous studies showed that a low capacity of the TCA cycle contributed to the Crabtree effect^44^, and a comprehensive analysis of omics data indicated that the TCA cycle flux was to some extent controlled by phosphorylation of pyruvate dehydrogenase and one mutation in the pyruvate dehydrogenase complex E1 α subunit Pda1p was found to bypass this regulation^45^. As part of the first strategy we therefore overexpressed both wild-type and mutated *PDA1 (PDA1 [S313A])* to drive more flux to L-ornithine synthesis. In addition, we also overexpressed other genes that are involved in the conversion of pyruvate to α-ketoglutarate, including one of the pyruvate carboxylase isomer-encoding gene *PYC2*, the citrate synthase-encoding gene *CIT1*, the aconitase-encoding gene *ACO2* and the isocitrate dehydrogenase-encoding gene *IDP1*. The strain M1cM2qM3a, carrying the overexpression of *PDA1*, *PYC2*, *CIT1*, *ACO2* and *IDP1*, had an L-ornithine titre of 245 mg l^−1^, representing a 28% increase compared with its parent strain M1cM2q (Fig. 4d). By contrast, overexpression of the mutated m*PDA1* together with overexpression of *PYC2*, *CIT1*, *ACO2*, *IDP1* (strain M1cM2qM3b) only resulted in a slight increase in the L-ornithine titre of 264 mg l^−1^ (Fig. 4d) compared with strain M1cM2qM3a. The strains M1cM2qM3b and M1cM2qM3a exhibited approximately twofold L-ornithine/glucose yield as compared with the parent, whereas there was no substantial difference when compared with the control strain in terms of ethanol yield, biomass yield and maximum specific growth rate (Supplementary Table 8). Thus, with this strategy the Crabtree effect was not substantially alleviated; however, the metabolic flux towards α-ketoglutarate was apparently improved to some extent.

We previously showed that the Crabtree effect is partly due to a limited capacity of the respiration chain and that overexpression of NADH alternative oxidase (AOX) partly alleviated the overflow to ethanol^32^. We also found that AOX overexpression resulted in upregulation of almost every step of the TCA cycle, which may be beneficial for increasing the production of TCA cycle-derived chemicals, and this was supported by a previous proteomics study of an AOX-overexpressing strain^46^. We therefore overexpressed AOX from *Hansenula anomala* (*HaAOX1*) and also *NDI1* encoding the endogenous NADH dehydrogenase, which mediates the delivery of electrons to the respiratory chain. Overexpression of *HaAOX1* (strain M1cM2qM3c) resulted in an L-ornithine titre of 258 mg l^−1^, representing a 35% increase as compared with the parent strain M1cM2q (Fig. 4d). Combined overexpression of *NDI1* and *HaAOX1* further increased L-ornithine production to 278 mg l^−1^ (Fig. 4d). These results indicated that overexpression of alternative NADH oxidase was an efficient strategy to boost the TCA cycle flux for the production of TCA cycle-derived amino acids. Interestingly, overexpression of *HaAOX1* and *NDI1* (strain M1cM2qM3d) increased L-ornithine production to the same level as boosting the TCA cycle's enzyme activity by direct overexpression of the related genes (strain M1cM2qM3b; Fig. 4d).

Although the two strategies described above had a positive effect on L-ornithine production, the Crabtree effect still caused low yields of L-ornithine on glucose, with ethanol being a major by-product. Previous work indicated a strong positive correlation between the Crabtree effect and glucose uptake rate during respirofermentative growth of *S. cerevisiae*^44^, and a recent study found that an internal deletion in the hexose transporter regulation protein Mth1p, encoded by *MTH1-ΔT*, interfered with its degradation and caused a decrease in expression of these transporter proteins and hence a reduced glucose uptake rate^47^. As the third strategy, we therefore overexpressed *MTH1-ΔT* (strain M1cM2qM3e) resulting in a remarkable fourfold increase in L-ornithine titre up to 778 mg l^−1^ (Fig. 4d). With *MTH1-ΔT* expression there was also no ethanol produced; however, the glucose uptake rate was reduced to 0.2 g glucose (g DCW)^−1 ^h^−1^, compared with the parent strain that had an ethanol production of 1.3 g ethanol (g DCW)^−1^ h^−1^ and a glucose uptake rate of 2.2 g glucose (g DCW)^−1 ^h^−1^ (Fig. 4e). These results demonstrated that alleviating the Crabtree effect of *S. cerevisiae* was an efficient strategy to boost L-ornithine production at high glucose concentrations.

Low activity of α-ketoglutarate dehydrogenase (ODC), which catalyses the oxidative decarboxylation of α-ketoglutarate to succinyl-CoA in the TCA cycle, was found to be essential for glutamate overproduction in *C. glutamicum*^48^, and, inspired by this, we reasoned that ODC attenuation might redirect more TCA flux to the biosynthesis of L-ornithine. However, deletion of *KGD2* encoding one of the components of the mitochondrial α-ketoglutarate dehydrogenase complex decreased the L-ornithine titre by ∼90% (Supplementary Fig. 16). Interestingly, the glucose uptake and the ethanol production rate of *KGD2* deletion strain (Strain M1dM2qM3f) increased to 2.2 g glucose (g DCW)^−1 ^h^−1^ and 0.9 g ethanol (g DCW)^−1 ^h^−1^, respectively (Supplementary Table 7).

Clearly alleviation of the Crabtree effect has a positive effect on the L-ornithine titre and yield. Even though overexpression of *MTH1-ΔT* was found to be an efficient strategy for alleviating the Crabtree effect in shake-flask cultures, it would be less attractive for industrial production as the strain has reduced growth. We therefore explored the possibility of alleviating the Crabtree effect by using an aerobic glucose-limited fed-batch strategy with strain M1cM2qM3a, whose upstream TCA cycle was enhanced as described above. This resulted in a final L-ornithine titre of 5.1 g l^−1^ and a final DCW of 56 g l^−1^ (Supplementary Fig. 17).

### Urea cycle engineering enables L-ornithine titre improvement

*S. cerevisiae* has the potential to operate the urea cycle: arginase (encoded by *CAR1*) can degrade L-arginine into urea and L-ornithine. To further increase L-ornithine synthesis and reduce the L-arginine pool, which can cause feedback inhibition, we also overexpressed *CAR1*. As expected, overexpression of *CAR1* in the strain M1cM2qM3e (resulting in strain M1dM2qM3e) enabled a final L-ornithine titre of 1,041 mg l^−1^, representing a further 34% increase. This final strain has a 23-fold improvement in L-ornithine production compared with strain M1c (Fig. 4d). An early study showed that in the presence of L-arginine and L-ornithine, OTC (Arg3p) and arginase (Car1p), both trimeric proteins, can form the so-called ‘epi-arginase' mode of regulation, that is, a one-to-one complex in which arginase remains active but OTC activity is inhibited^31^. This regulation prevents the formation of an L-ornithine futile cycle when the biosynthesis of L-arginine is repressed and the catabolism of L-arginine is activated^31^. We speculate that overexpression of *CAR1* also possibly improved the ‘epi-arginase' efficiency and the flux from L-ornithine to L-arginine was well controlled at a moderate level.

## Discussion

Metabolic engineering in yeast is often hampered by extensive regulation of its metabolism, which evolved to ensure robustness. Extensive genetic perturbations therefore have to be implemented in a coordinated manner to achieve high-level production of desired chemicals, especially pathway intermediates. We demonstrated that a MPR strategy enabled a coordinated increase in flux towards L-ornithine. The MPR approach involved implementation of a large number of perturbations in the metabolism, ranging from the central carbon metabolism to the dedicated amino-acid biosynthetic pathway. Here we used a serial approach; however, recent advances in molecular biology tools for yeast and other eukaryotic organisms^15^,^17^,^49^,^50^ will enable concurrent tinkering of the many different pathway modules considered here.

Enhancement of the precursor supply is a very common strategy in bacteria to increase the flux towards the desired amino acids^13^,^43^,^51^. Yet, the segmentation of precursor intermediates in different subcellular organelles in *S. cerevisiae* hampers the plug and play use of such approaches^52^,^53^. Thus, we tested a serial of customized approaches for yeast to improve precursor supply for L-ornithine synthesis. When we implemented Module 2, a substantial increase in L-ornithine titre was observed when the mitochondrial trafficking steps of L-ornithine and L-glutamate were enhanced by overexpressing the related proteins belonging to the mitochondrial carrier family, suggesting the mitochondrial subcellular trafficking often to be flux-controlling steps. However, when we implemented the overexpression of *ODC1*, which was suggested to encode one of the transporters responsible for the export of α-oxoglutarate and α-oxoadipate from the mitochondrial matrix to the cytosol, where they are required for glutamate and lysine biosyntheses, the L-ornithine titre did not substantially increase. This could be due to a low level of α-ketoglutamate in the mitochondria; however, it may also be such that Odc1p is not a specific transporter responsible for the efflux of α-ketoglutamate from the mitochondrial matrix to the cytosol. Thus, while the identification of new mitochondrial carriers including the said α-ketoglutamate mitochondrial transporter in yeast will provide new insights into the physiological roles of mitochondrial carriers in cell metabolism, it would also allow for improved metabolic engineering.

In other studies, pathway re-localization was shown to be a successful strategy to improve the production efficiency of desired chemicals^11^,^20^,^54^. Owing to the concentration of enzymes and intermediates, reduction in toxic effects of certain intermediates and prevention in the diversion of precursor flux into competing pathways^11^,^53^,^54^, re-localization of the metabolic pathways to the mitochondria was shown to be a useful strategy in metabolic engineering^11^,^53^,^54^. However, in contrast to the substantial increase in L-ornithine titre when we introduced the bacterial acetylated derivatives cycle into the cytoplasm, targeting NADPH-dependent glutamate dehydrogenase (Gdh1p) to the mitochondria markedly decreased the L-ornithine titre. These results highlight the importance of carefully choosing the re-localization strategy for metabolic engineering, and our results demonstrate that it may be better to express a heterologous bacterial pathway in the cytosol than to de-regulate the endogenous mitochondrial pathway. Similarly, one recent work showed that cytosolic pathway re-localization increased isobutanol production substantially in *S. cerevisiae* and also showed that further blocking of the parallel mitochondrial L-valine pathway, which generated the driving force for the cytosolic isobutanol pathway, resulted in even higher isobutanol production^20^.

Although iterative engineering improved L-ornithine production to 192 mg l^−1^ in strain M1cM2q, the L-ornithine yield on glucose was only 9.6 mg (g glucose)^−1^; moreover, several studies reported similarly low yields on the carbon substrate^21^,^22^,^23^ after metabolic engineering of *S. cerevisiae* for production of chemicals, especially amino-acid-derived molecules. One of the main reasons is the Crabtree effect by which substantial carbon flux is redirected to ethanol production^44^,^55^,^56^. Through optimization of Module 3, we showed that several different strategies worked and overexpression of a truncated form of *MTH1*, that is, *MTH1-ΔT*, was the most successful one for increasing the L-ornithine yield as it generally alleviates the Crabtree effect even when the cells are grown at high glucose concentrations. However, the reduced maximum specific growth rate compared with that of the parent strain makes this strategy less attractive for large-scale process, where the Crabtree effect can be avoided through the use of fed-batch fermentations as also demonstrated here. Still, overexpression of *MTH1-ΔT* for reducing the substrate uptake and the associated overflow metabolism is here demonstrated to be a useful strategy for evaluation of engineering strategies in shake-flask cultures.

In conclusion, despite the complexity of the L-ornithine biosynthetic pathway, our results demonstrate great promise for engineering of yeast for production of amino acids using the MPR approach. The L-ornithine biosynthesis and regulation, pathway compartmentalization and overflow metabolism were systematically modified for L-ornithine overproduction. This first successful proof-of-concept of L-ornithine production in *S. cerevisiae* showed the potential to engineer other fungal microbial cell factories for production of amino acids and amino-acid-derived chemicals. In addition, our engineered strains and strategies described here should facilitate future engineering of complex biosynthetic pathways for production of different natural products derived from L-ornithine and even other amino acids intermediates.

## Methods

### Yeast strains and plasmids

All engineered strains (Supplementary Table 1 and Supplementary Table 2) were constructed from *S. cerevisiae* strain CEN.PK 113-11C (*MAT*a *SUC2 MAL2-8c his3Δ1 ura3-52)*. Genetic engineering based on plasmids or chromosomal integration was performed following ‘DNA assembler'^18^ and ‘MOPE strategy'^4^. Detailed pathway construction procedures including promoter replacement, chromosome integration and plasmid construction are described in the Supplementary Methods and Supplementary Tables 3, 5 and 6. All PCR primers used are listed in Supplementary Table 4.

### Media

Yeast strains without plasmids were maintained on YPD plates (10 g l^−1^ yeast extract, 20 g l^−1^ casein peptone, 20 g l^−1^ glucose and 20 g l^−1^ agar). Plasmid carrying yeast strains were selected on synthetic dextrose (SD) plates (6.7 g l^−1^ yeast nitrogen base (amino acids free), 20 g l^−1^ glucose and 20 g l^−1^ agar) with uracil and histidine omitted when appropriate. Defined minimal medium (Delft medium)^37^ was used for both batch cultivations and fed-batch fermentations of L-ornithine-producing strains. Detailed procedures of both shake-flask batch fermentation and fermenter fed-batch fermentation can be found in the Supplementary Methods.

### Cell dry weight measurement

The cell dry weight was measured by filtering known volumes of the cultures through pre-dried and pre-weighed 0.45-μm-pore size nitrocellulose filters. The filters with the biomass were washed with water, dried for 15 min in a microwave oven at 150 W and weighed again. The optical density at 600 nm was determined using a Hitachi U-1100 spectrophotometer. For the intracellular concentration estimation, the cell volume (*V*_C_) was calculated to *V*_C_=2.38 ml (g CDW)^−1^ (ref. 57).

### Extracellular metabolite detection using HPLC

Concentrations of glucose and ethanol were analysed by an isocratic high-performance liquid chromatograph (UltiMate 3000 Nano, Dionex) with an Aminex HPX-87H ion-exchange column (Bio-Rad, Hercules, CA) at 45 °C, and 5 mM H_2_SO_4_ as the mobile phase at a flow rate of 0.6 ml min^−1^. Glucose and ethanol were measured with a refraction index detector (RI-101 Refractive Index Detector, Shodex).

### L-arginine and L-ornithine extraction and quantification

Leakage-free quenching was performed before exaction according to ref. 58. Intracellular L-arginine and L-ornithine were extracted by using the Hot Water Extraction protocol, which was modified and performed according to ref. 58 with minor modifications. Then, both extracellular and intracellular L-arginine detections were performed with an L-arginine rapid detection Kit (Megazyme K-LARGE, Wicklow, Ireland), and L-ornithine was measured using a ninhydrin colorimetric assay as described previously^59^.

### L-ornithine qualitative analysis with GC–MS

The analysis of extracellular L-ornithine samples was performed according to ref. 60. Briefly, L-ornithine was derivatized by tert-butyldimethylsilylation and further analysed with GC–MS. Confirmation of the identity of L-ornithine from the sample was achieved by comparing its retention time and mass spectrum with the synthetic standard under identical measurement conditions.

## Additional information

**How to cite this article:** Qin, J. *et al.* Modular pathway rewiring of *Saccharomyces cerevisiae* enables high-level production of L-ornithine. *Nat. Commun.* 6:8224 doi: 10.1038/ncomms9224 (2015).

## Supplementary Material

Supplementary Information Supplementary Figures 1-17, Supplementary Tables 1-8, Supplementary Methods and Supplementary References.

## Figures and Tables

**Table 1:** Main strains used for module and full pathway optimization

**No.**	**Strains**	**Module 1**		**Module 2**		**Module 3**	
		**Module**	**Description**	**Module**	**Description**	**Module**	**Description**
1	M1a	M1a	*P*_*HXT1*_*-ARG3*	Null	Null	Null	Null
2	M1b	M1b	*P*_*KEX2*_*-ARG3*	Null	Null	Null	Null
3	M1c	M1c	*P*_*KEX2*_*-ARG3; car2Δ*	Null	Null	Null	Null
4	M1cM2a	M1c	*P*_*KEX2*_*-ARG3; car2Δ*	M2a	*ARG2*	Null	Null
5	M1cM2b	M1c	*P*_*KEX2*_*-ARG3; car2Δ*	M2b	*ARG5,6*	Null	Null
6	M1cM2c	M1c	*P*_*KEX2*_*-ARG3; car2Δ*	M2c	*ARG7*	Null	Null
7	M1cM2d	M1c	*P*_*KEX2*_*-ARG3; car2Δ*	M2d	*MLS-argB*_*Cg*_	Null	Null
8	M1cM2e	M1c	*P*_*KEX2*_*-ARG3; car2Δ*	M2e	*MLS-argJ*_*Cg*_	Null	Null
9	M1cM2f	M1c	*P*_*KEX2*_*-ARG3; car2Δ*	M2f	*ARG5,6; ARG7; ARG8*	Null	Null
10	M1cM2g	M1c	*P*_*KEX2*_*-ARG3; car2Δ*	M2g	*ARG5,6; ARG7; ARG8; ARG2*	Null	Null
11	M1cM2h	M1c	*P*_*KEX2*_*-ARG3; car2Δ*	M2h	*ARG5,6; ARG7; ARG8; ARG2; ORT1*	Null	Null
12	M1cM2i	M1c	*P*_*KEX2*_*-ARG3; car2Δ*	M2i	*ARG5,6; ARG7; ARG8; ARG2; ORT1; MLS-GDH1*	Null	Null
13	M1cM2j	M1c	*P*_*KEX2*_*-ARG3; car2Δ*	M2j	*ARG5,6; ARG7; ARG8; ARG2; ORT1; MLS-GDH2*	Null	Null
14	M1cM2k	M1c	*P*_*KEX2*_*-ARG3; car2Δ*	M2k	*ARG5,6; ARG7; ARG8; ARG2; ORT1; AGC1*	Null	Null
15	M1cM2l	M1c	*P*_*KEX2*_*-ARG3; car2Δ*	M2l	*ARG5,6; ARG7; ARG8; ARG2; ORT1; AGC1; GDH1*	Null	Null
16	M1cM2m	M1c	*P*_*KEX2*_*-ARG3; car2Δ*	M2m	*ARG5,6; ARG7; ARG8; ARG2; ORT1; AGC1; GDH3*	Null	Null
17	M1cM2n	M1c	*P*_*KEX2*_*-ARG3; car2Δ*	M2n	*ARG5,6; ARG7; ARG8; ARG2; ORT1; AGC1; GLN1; GLT1*	Null	Null
18	M1cM2o	M1c	*P*_*KEX2*_*-ARG3; car2Δ*	M2o	*ARG5,6; ARG7; ARG8; ARG2; ORT1; AGC1; GDH3; ODC1*	Null	Null
19	M1cM2p	M1c	*P*_*KEX2*_*-ARG3; car2Δ*	M2p	*ARG5,6; ARG7; ARG8; ARG2; ORT1; AGC1; GDH1; ODC1*	Null	Null
20	M1cM2q	M1c	*P*_*KEX2*_*-ARG3; car2Δ*	M2q	*argA*_*Ec;*_*argB*_*Ec*_*; argC*_*Cg*_*; argD*_*Cg*_*; argJ*_*Cg*_*; ORT1; AGC1; GDH1*	Null	Null
21	M1cM2r	M1c	*P*_*KEX2*_*-ARG3; car2Δ*	M2r	*P*_*ADH1*_*- tGCN4*	Null	Null
22	M1cM2s	M1c	*P*_*KEX2*_*-ARG3; car2Δ*	M2s	*P*_*TEF1*_*- tGCN4*	Null	Null
23	M1cM2t	M1c	*P*_*KEX2*_*-ARG3; car2Δ*	M2t	*P*_*GPD1*_*- tGCN4*	Null	Null
24	M1cM2qM3a	M1c	*P*_*KEX2*_*-ARG3; car2Δ*	M2q	*argA*_*Ec;*_*argB*_*Ec*_*; argC*_*Cg*_*; argD*_*Cg*_*; argJ*_*Cg*_*; ORT1; AGC1; GDH1*	M3a	*PDA1;CIT1;ACO2;IDP1;PYC2*
25	M1cM2qM3b	M1c	*P*_*KEX2*_*-ARG3; car2Δ*	M2q	*argA*_*Ec;*_*argB*_*Ec*_*; argC*_*Cg*_*; argD*_*Cg*_*; argJ*_*Cg*_*; ORT1; AGC1; GDH1*	M3b	*PDA1[S313A];CIT1;ACO2;IDP1;PYC2*
26	M1cM2qM3c	M1c	*P*_*KEX2*_*-ARG3; car2Δ*	M2q	*argA*_*Ec;*_*argB*_*Ec*_*; argC*_*Cg*_*; argD*_*Cg*_*; argJ*_*Cg*_*; ORT1; AGC1; GDH1*	M3c	*HaAOX1*
27	M1cM2qM3d	M1c	*P*_*KEX2*_*-ARG3; car2Δ*	M2q	*argA*_*Ec;*_*argB*_*Ec*_*; argC*_*Cg*_*; argD*_*Cg*_*; argJ*_*Cg*_*; ORT1; AGC1; GDH1*	M3d	*HaAOX1; NDI1*
28	M1cM2qM3e	M1c	*P*_*KEX2*_*-ARG3; car2Δ*	M2q	*argA*_*Ec;*_*argB*_*Ec*_*; argC*_*Cg*_*; argD*_*Cg*_*; argJ*_*Cg*_*; ORT1; AGC1; GDH1*	M3e	*MTH1-ΔT*
29	M1cM2qM3f	M1c	*P*_*KEX2*_*-ARG3; car2Δ*	M2q	*argA*_*Ec;*_*argB*_*Ec*_*; argC*_*Cg*_*; argD*_*Cg*_*; argJ*_*Cg*_*; ORT1; AGC1; GDH1*	M3f	*MTH1-ΔT; kgd2Δ*
30	M1dM2q	M1d	*P*_*KEX2*_*-ARG3; car2Δ*	M2q	*argA*_*Ec;*_*argB*_*Ec*_*; argC*_*Cg*_*; argD*_*Cg*_*; argJ*_*Cg*_*; ORT1; AGC1; GDH1*	Null	Null
31	M1dM2qM3c	M1d	*P*_*KEX2*_*-ARG3; car2Δ*	M2q	*argA*_*Ec;*_*argB*_*Ec*_*; argC*_*Cg*_*; argD*_*Cg*_*; argJ*_*Cg*_*; ORT1; AGC1; GDH1*	M3c	*HaAOX1*
32	M1dM2qM3e	M1d	*P*_*KEX2*_*-ARG3; car2Δ*	M2q	*argA*_*Ec;*_*argB*_*Ec*_*; argC*_*Cg*_*; argD*_*Cg*_*; argJ*_*Cg*_*; ORT1; AGC1; GDH1*	M3e	*MTH1-ΔT*
33	M1dM2qM3f	M1d	*P*_*KEX2*_*-ARG3; car2Δ*	M2q	*argA*_*Ec;*_*argB*_*Ec*_*; argC*_*Cg*_*; argD*_*Cg*_*; argJ*_*Cg*_*; ORT1; AGC1; GDH1*	M3f	*MTH1-ΔT; kgd2Δ*
34	B0166A (ORT1)	Null	Null	Null	Null	Null	Null

Strain names indicate the modules present in the strain, for example, M1aM2bM3c includes strategy a for Module 1, strategy b for Module 2 and strategy c for Module 3.
