# Supplementary material for: Modular pathway rewiring of Saccharomyces cerevisiae enables high-level production of L-ornithine
Source: Nat Commun. 2015 Sep 8;6:8224. doi: 10.1038/ncomms9224 (PMC4569842; doi:10.1038/ncomms9224)
Supplement: Supplementary Information — Supplementary Figures 1-17, Supplementary Tables 1-8, Supplementary Methods and Supplementary References. [file ncomms9224-s1.pdf]

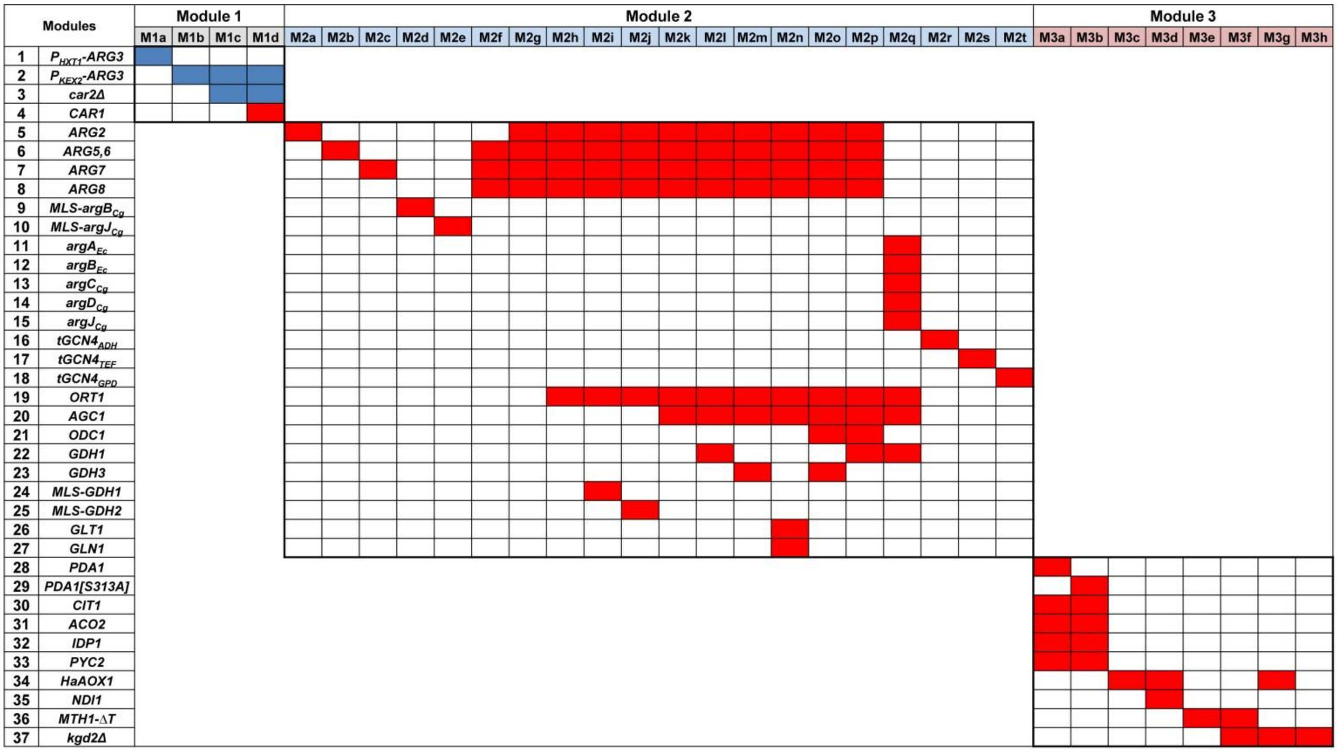

Supplementary Figure 1| All targets subject to perturbation in the Modular Pathway Rewiring (MPR). *CAR1*, arginase; *CAR2*, L-ornithine transaminase; *ARG3*, ornithine carbamoyltransferase; *ARG2*, glutamate N-acetyltransferase; *ARG5,6*, acetylglutamate kinase and N-acetyl-gamma-glutamyl-phosphate reductase; *ARG7*, mitochondrial ornithine acetyltransferase; *ARG8*, acetylornithine aminotransferase; *MLS-argJ*$_{Cg}$, ornithine acetyltransferase from *C. glutamicum* located in the mitochondria of *S. cerevisiae*; *MLS-argB*$_{Cg}$, acetylglutamate kinase from *C. glutamicum* located in the mitochondria of *S. cerevisiae*; *argA*$_{Ec}$, glutamate N-acetyltransferase from *E .coli*; *argB*$_{Ec}$, acetylglutamate kinase from *E. coli*; *argC*$_{Cg}$, N-acetyl-gamma-glutamyl-phosphate reductase from *C. glutamicum*; *argD*$_{Cg}$, acetylornithine aminotransferase from *C. glutamicum;* *argJ*$_{Cg}$, ornithine acetyltransferase from *C. glutamicum*; *ORT1*, ornithine transporter of the mitochondrial inner membrane; *AGC1*, glutamate uniporter ; *ODC1*, transporter of α-ketodicarboxylate or α-ketoglutarate of the mitochondrial inner membrane; *GDH1*, $NADP^+$-dependent glutamate dehydrogenase; *GDH3*, $NADP^+$-dependent glutamate dehydrogenase; *GLT1*, NAD^+-dependent glutamate synthase; *GLN1*, glutamine synthetase; *GDH2*, NAD^+-dependent glutamate dehydrogenase; *MLS-GDH1*, mitochondrially targeted $NADP^+$-dependent glutamate dehydrogenase; *MLS-GDH2*, mitochondrially targeted NAD^+-dependent glutamate dehydrogenase; *CIT1*, citrate synthase; *PYC2*, pyruvate carboxylase isoform; *ACO2*, putative mitochondrial aconitase isozyme; *IDP1*, mitochondrial $NADP^+$-specific isocitrate dehydrogenase; *PDA1*, E1 alpha subunit of the pyruvate dehydrogenase (PDH) complex; *mPDA1*, *PDA1* with mutation S313A; *KGD2*, dihydrolipoyl transsuccinylase; *HcAOX1*, NADH alternative oxidase from *Hansenula anomala*; *MTH1-ΔT*, truncated version of *MTH1* which encodes a negative regulator of the glucose-sensing signal transduction pathway (225 bp internal deletion spanning from position 169 to 393 is implemented); *NDI1*, NADH:ubiquinone oxidoreductase.

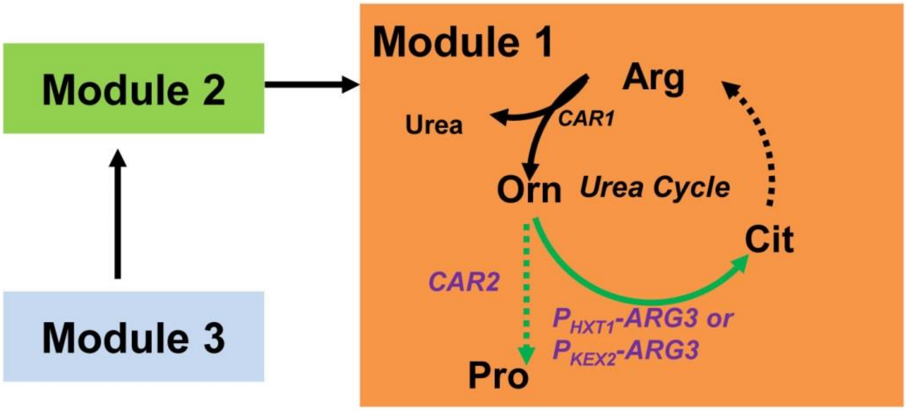

Supplementary Figure 2| The pathway overview of Module 1. The Modular Pathway Rewiring (MPR) was initiated from Module 1. *CAR1*, arginase; *CAR2*, L-ornithine transaminase; *ARG3*, ornithine carbamoyltransferase. Genes or proteins subject to over-expression are shown in red font, while those subject to attenuation or full-deactivation are shown in purple font. Solid thick arrows represent single reaction step, while the dashed black arrows represent multiple reaction steps. In addition, the dashed green arrow represents the first step in the L-ornithine degradation pathway to L-prolinesubject to gene deletion. P_{HXT1}-*ARG3*, the down-regulation of *ARG3* is implemented by replacing the original promoter of *ARG3* with the *HXT1* promoter. P_{KEX2}-*ARG3*, the down-regulation of *ARG3* is implemented by replacing the original promoter of *ARG3* with the *KEX2* promoter. See Figure 1 legend regarding abbreviations of metabolites.

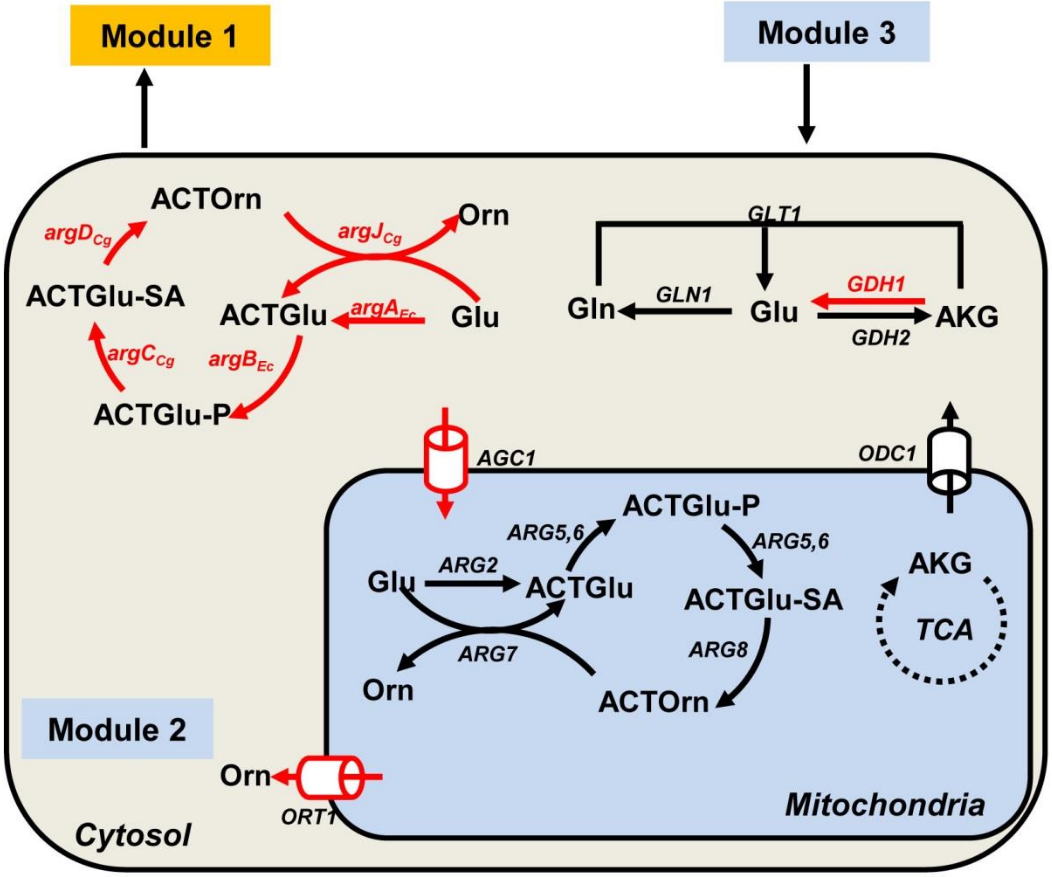

Supplementary Figure 3| The pathway overview of Module 2. Fonts and arrows are as described in Figure 1 legend. *ARG2*, glutamate N-acetyltransferase; *ARG5,6*, acetylglutamate kinase and N-acetyl-gamma-glutamyl-phosphate reductase; *ARG7*, mitochondrial ornithine acetyltransferase; *ARG8*, acetylornithine aminotransferase; *MLS-argJ*$_{Cg}$, ornithine acetyltransferase from *C. glutamicum* located in the mitochondria of *S. cerevisiae*; *MLS-argB*$_{Cq}$, acetylglutamate kinase from *C. glutamicum* located in the mitochondria of *S. cerevisiae*; *argA*$_{Ec}$, glutamate N-acetyltransferase from *E. coli*; *argB*$_{Ec}$, acetylglutamate kinase from *E. coli*; *argC*$_{Cq}$, N-acetyl-gamma-glutamyl-phosphate reductase from *C. glutamicum*; *argD*$_{Cq}$, acetylornithine aminotransferase from *C. glutamicum; argJ*$_{Cq}$, ornithine acetyltransferase from *C. glutamicum*; *ORT1*, ornithine transporter of the mitochondrial inner membrane; *AGC1*, glutamate uniporter ; *ODC1*, transporter of α-ketodicarboxylate or α-ketoglutarate of the mitochondrial inner membrane; *GDH1*, $NADP^+$-dependent glutamate dehydrogenase; *GDH3*, $NADP^+$-dependent glutamate dehydrogenase; *GLT1*, NAD^+-dependent glutamate synthase; *GLN1*, glutamine synthetase; *GDH2*, NAD^+-dependent glutamate dehydrogenase; *MLS-GDH1*, mitochondrially targeted $NADP^+$-dependent glutamate dehydrogenase; *MLS-GDH2*, mitochondrially targeted NAD^+-dependent glutamate dehydrogenase. See Figure 1 legend regarding abbreviations of metabolites.

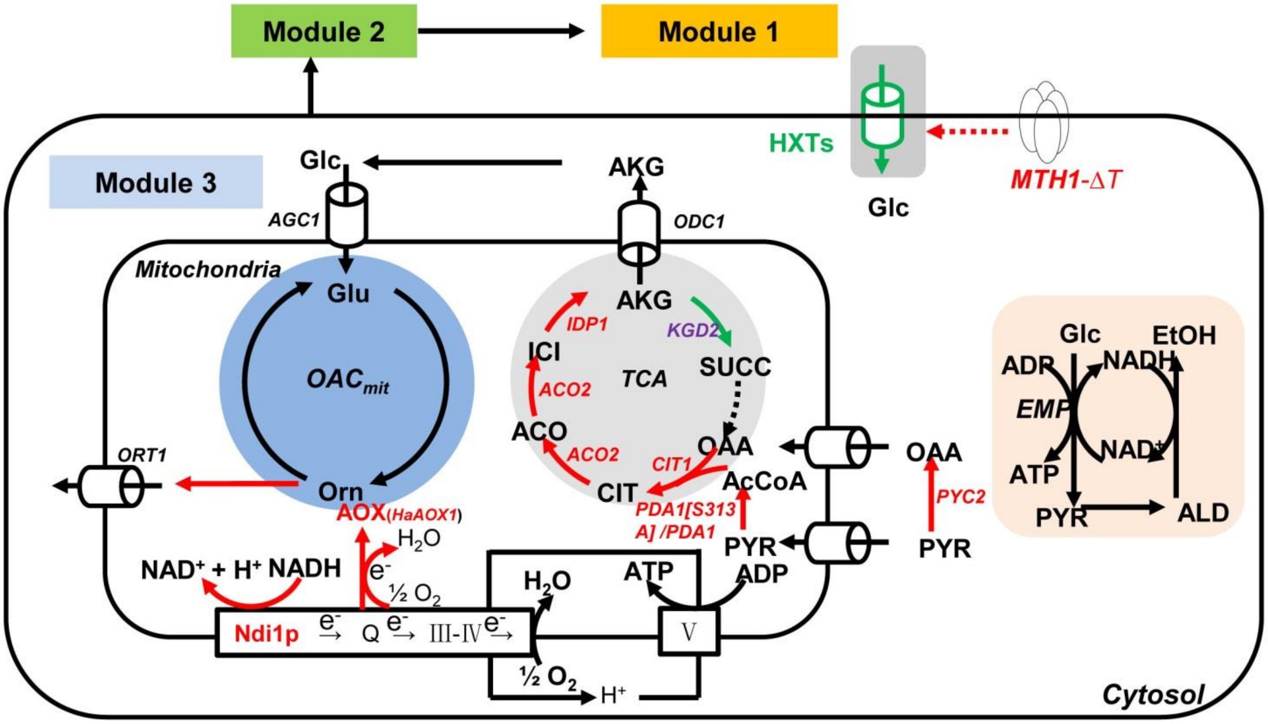

Supplementary Figure 4| The pathway overview of Module 3. Fonts and arrows are as described in Figure 1 legend. *ORT1*, ornithine transporter of the mitochondrial inner membrane; *AGC1*, glutamate uniporter ; *ODC1*, transporter of α-ketodicarboxylate or α-ketoglutarate of the mitochondrial inner membrane; *CIT1*, citrate synthase; *PYC2*, pyruvate carboxylase isoform; *ACO2*, putative mitochondrial aconitase isozyme; *IDP1*, mitochondrial $NADP^+$-specific isocitrate dehydrogenase; *PDA1*, E1 alpha subunit of the pyruvate dehydrogenase (PDH) complex; *PDA1[S313A]*, *PDA1* with mutation S313A; *KGD2*, dihydrolipoyl transsuccinylase; *HaAOX1*, NADH alternative oxidase from *H. anomala*; *MTH1*-Δ*T*, truncated version of *MTH1* which encodes a negative regulator of the glucose-sensing signal transduction pathway (225 bp internal deletion spanning from position 169 to 393 is implemented); Ndi1p, NADH:ubiquinone oxidoreductase. See Figure 1 legend regarding other abbreviations.

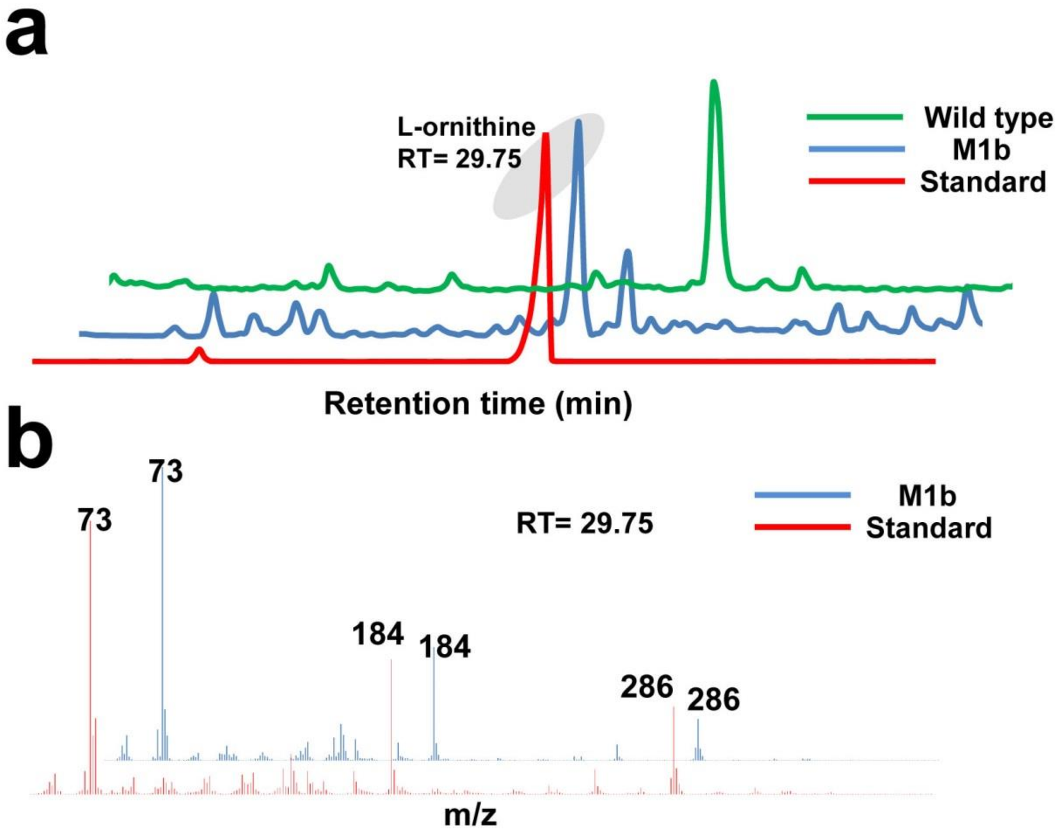

Supplementary Figure 5| Qualitative verification of L-ornithine produced by proof of concept strain with GC-MS. (a) Gas chromatogram of L-ornithine (retention time 29.75 min). (b) High resolution mass spectrometry of L-ornithine.

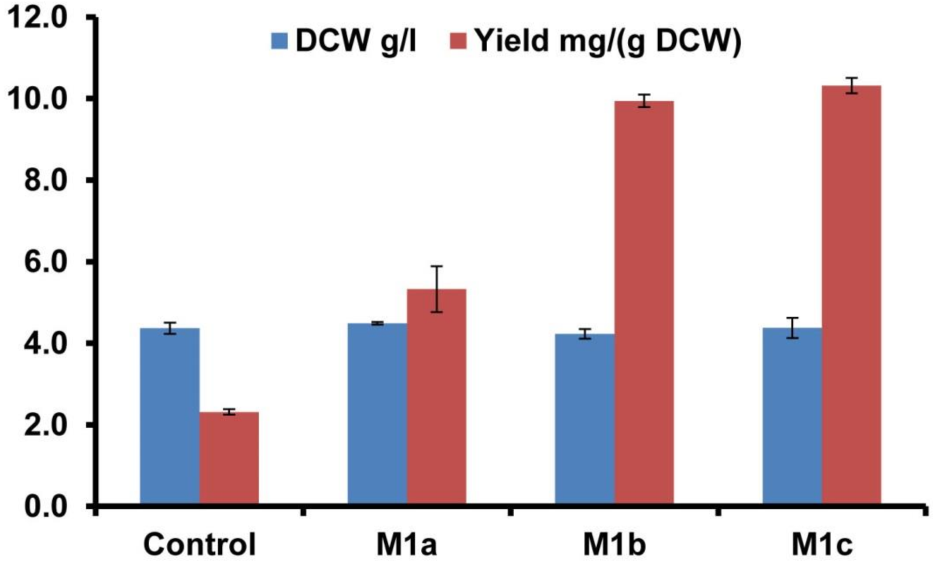

Supplementary Figure 6| Leaky L-arginine auxotrophy enables L-ornithine over-production (Module 1). Both DCW and L-ornithine yield normalized to DCW are shown.

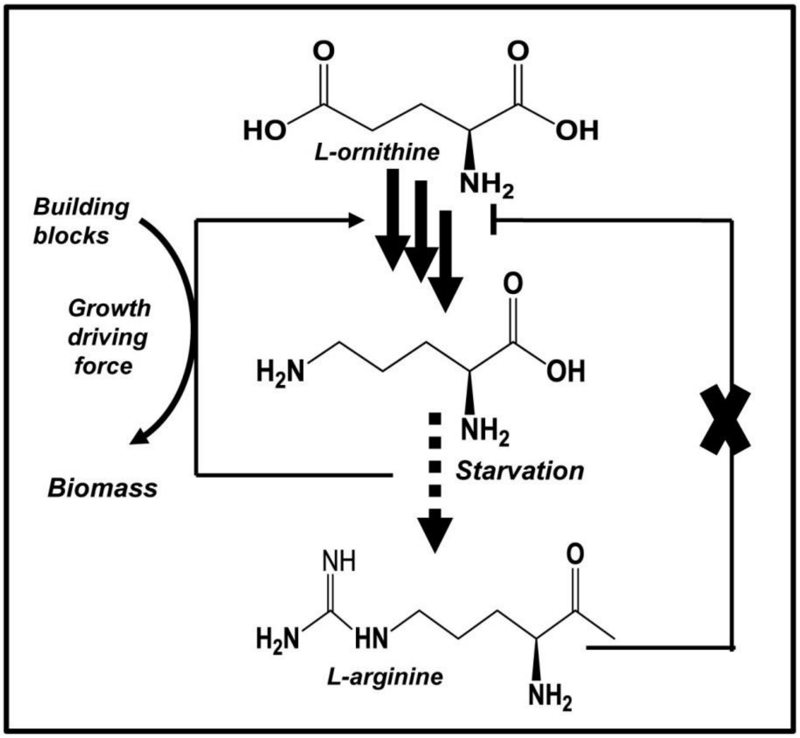

Supplementary Figure 7| Proposed underlying mechanisms by which the L-ornithine 'acetylated derivatives cycle' flux was boosted when *ARG3* was down-regulated.

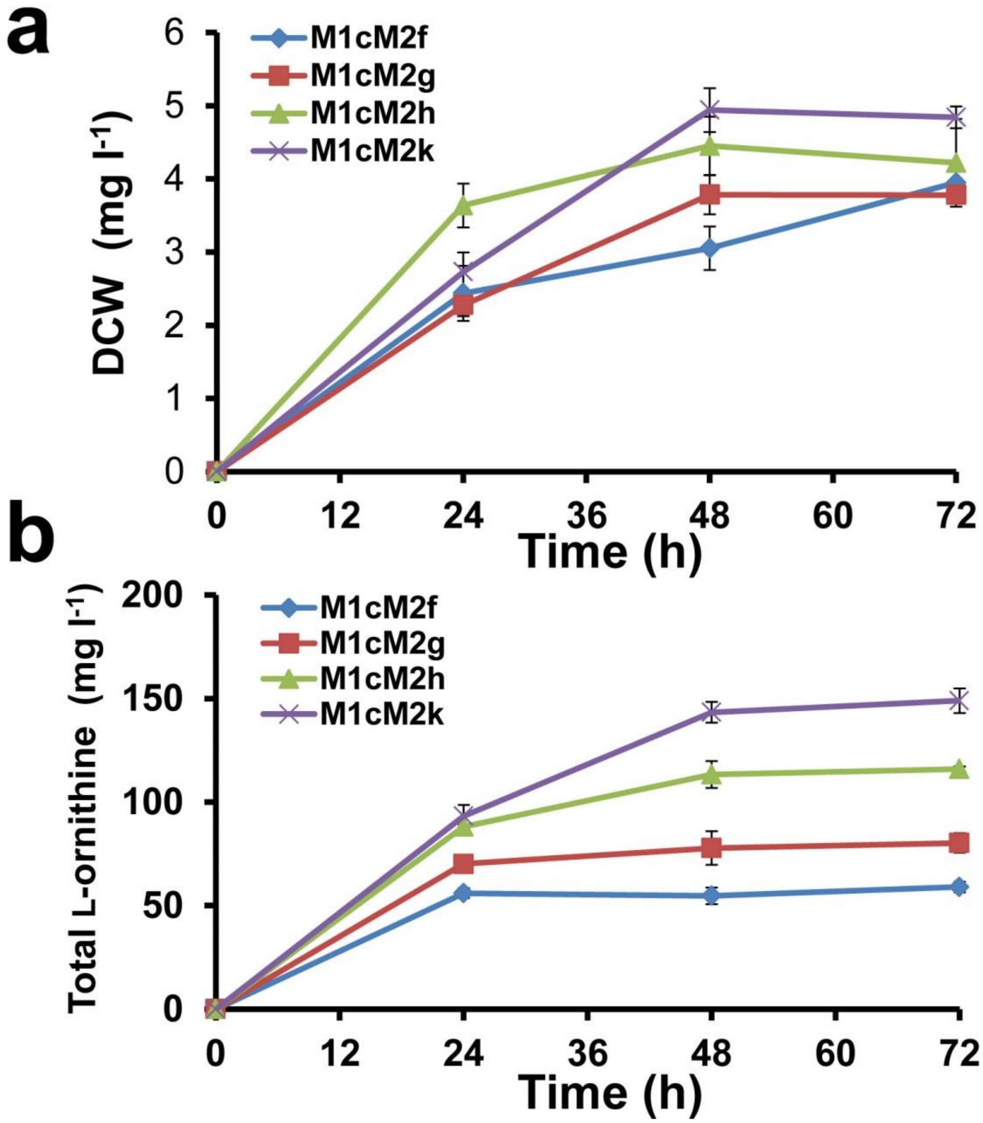

Supplementary Figure 8| Typical time profiles of cell growth and L-ornithine production of engineered strains. Cells (strains M1cM2f, M1cM2g, M1cM2h and M1cM2k) were grown in defined minimal medium with 20 g l^{-1} glucose and cultures were sampled after 24 h, 48 h and 72 h of growth for L-ornithine titer (b) and biomass (a) determination. Displayed are the average values ± standard deviation from three biological replicates.

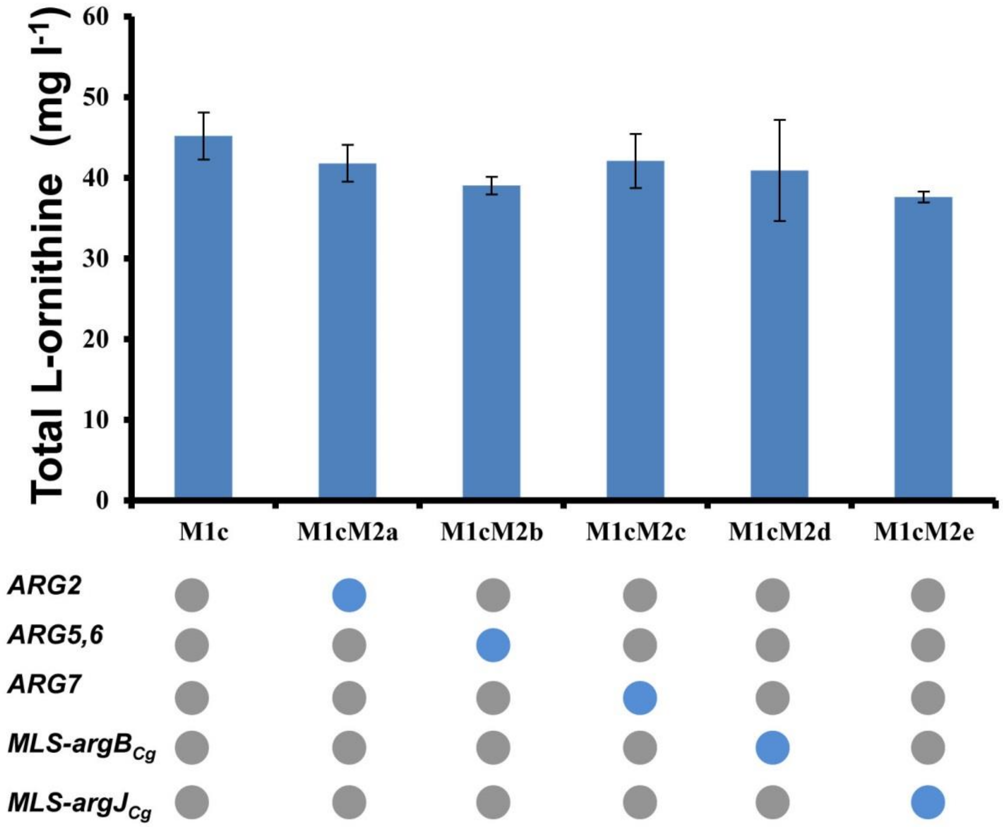

Supplementary Figure 9| The single overexpression of genes in the L-ornithine 'acetylated derivatives cycle' showed no substantial effect on the L-ornithine titer. All strains were cultivated for 72 h in Delft medium. All data are presented as the mean ± s.d. (n≥3).

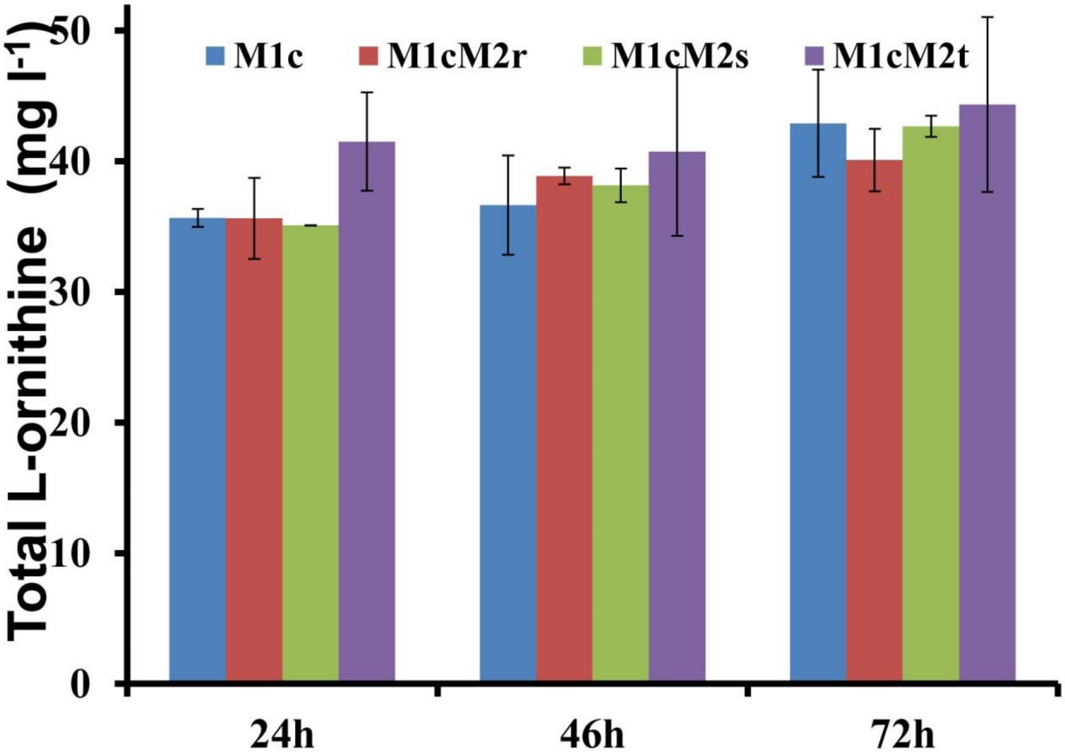

Supplementary Figure 10| The effect of truncated Gcn4p overexpression on L-ornithine titer. All strains were cultivated for 72 h in Delft medium. All data are presented as the mean ± s.d. (n≥3).

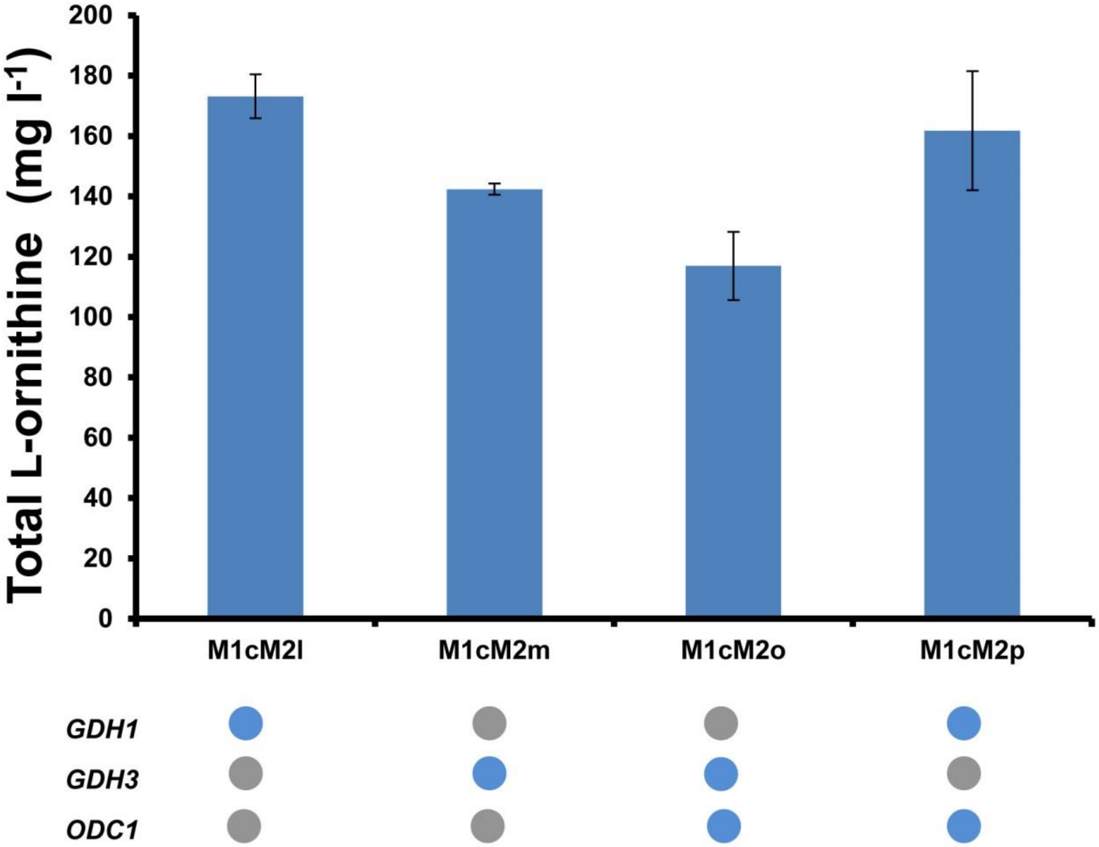

Supplementary Figure 11| The effect of *ODC1* overexpression of L-ornithine titre in both *GDH1* and *GDH3* overexpression strains. All strains were cultivated for 72 h in Delft medium. All data are presented as the mean ± s.d. (n≥3).

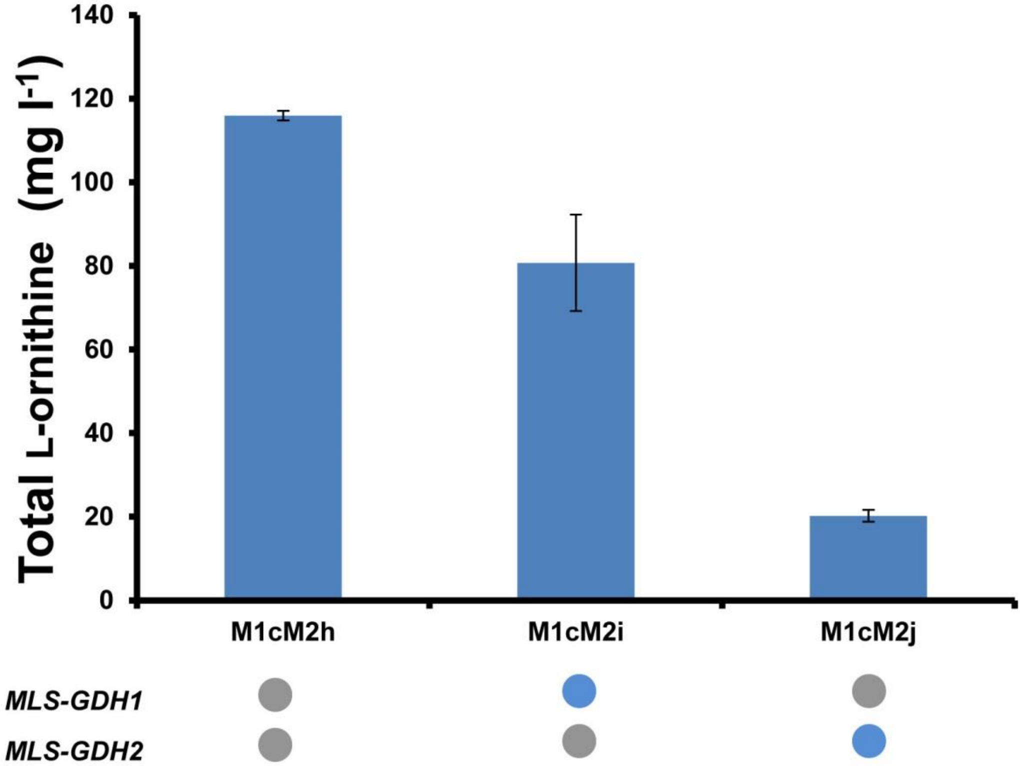

Supplementary Figure 12| Mitochondrial targeting of *GDH1* and *GDH2* substantially decreased L-ornithine titres. All strains were cultivated for 72 h in Delft medium. All data are presented as the mean ± s.d. (n≥3).

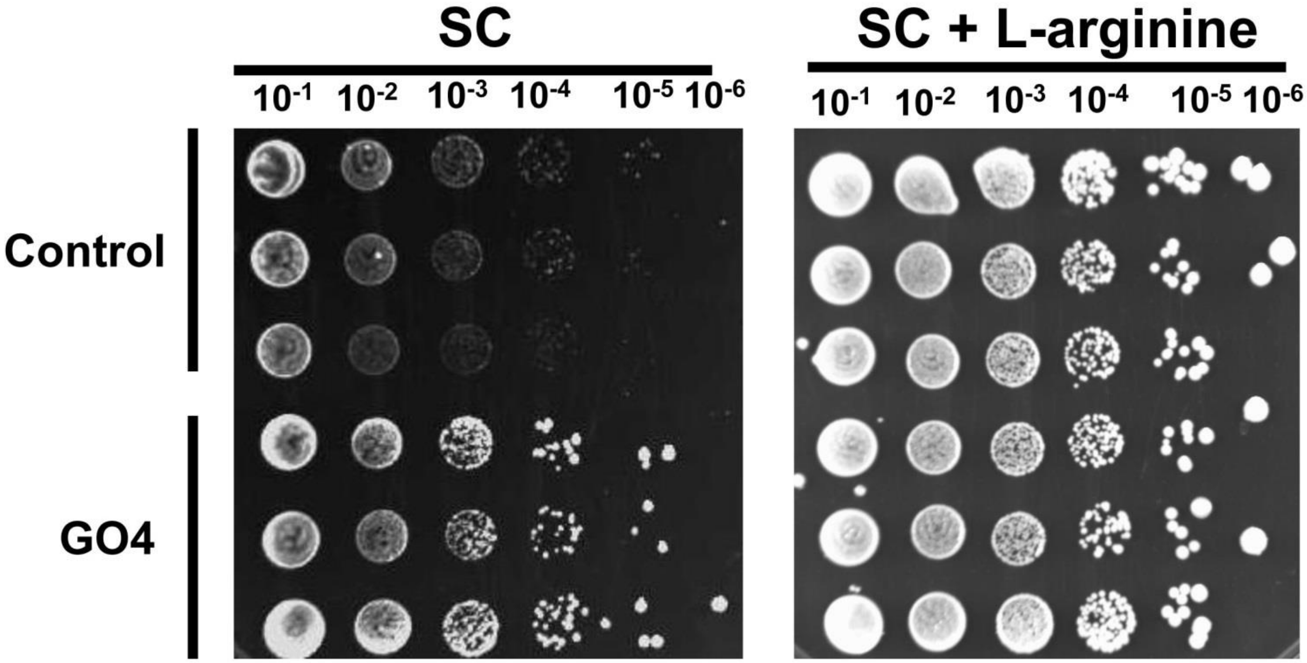

Supplementary Figure 13| The cytosolic L-ornithine synthesis pathway was functionally verified by complementation. While the control strain (*ort1*Δ) grows poorly in the medium without L-arginine, the strain harboring the cytosolic pathway restored the ability to grow on L-arginine negative medium. Control represents strain B0166A (p423GPD) and GO4 represents strain B0166A (ORT1) expressing the synthetic cytoplasmic L-ornithine pathway. The overnight cultured cells were diluted to an OD600 of 1, and 5 μl aliquots of dilutions from 10^{-1} to10^{-6} were spotted on the corresponding plates.

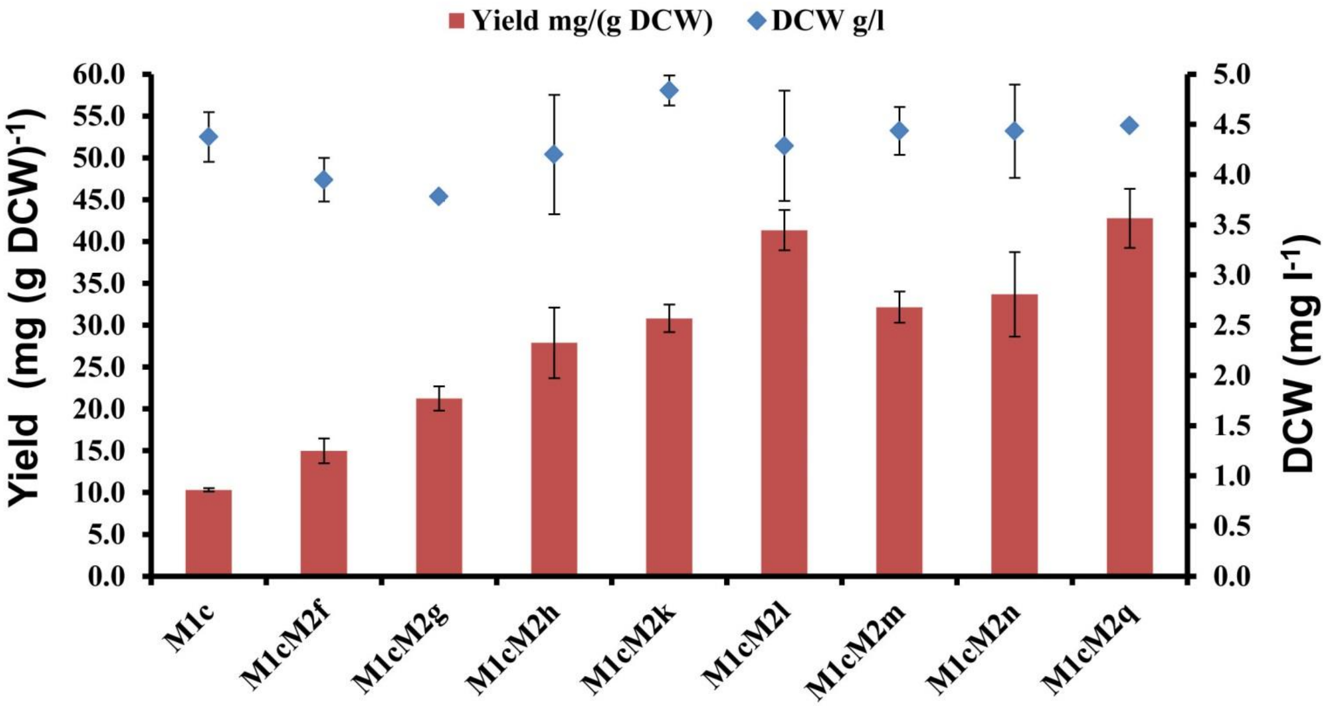

Supplementary Figure 14| Subcellular trafficking engineering and pathway translocation elevates L-ornithine synthesis. Both DCW and L-ornithine yield normalized to DCW are shown.

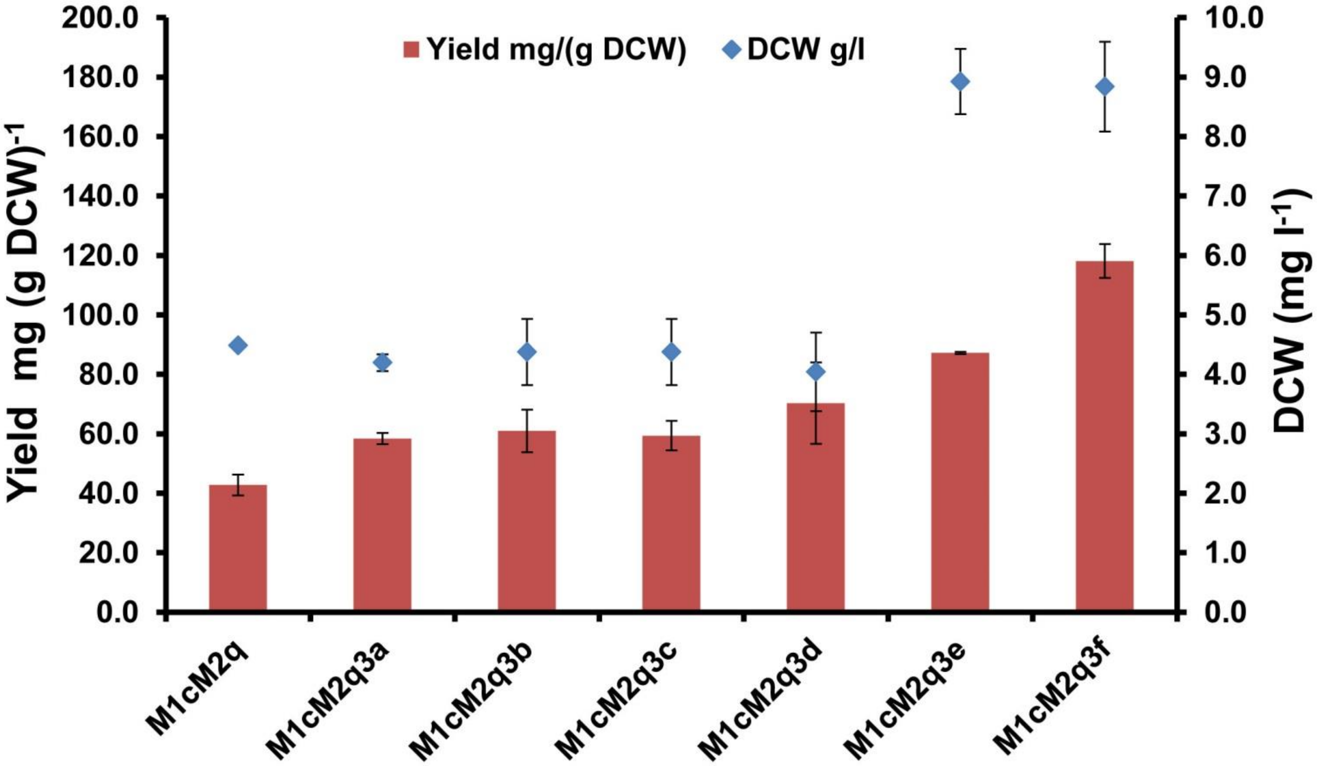

Supplementary Figure 15| Attenuation of the 'Crabtree effect' improves carbon channeling to L-ornithine (Module 3). Both DCW and L-ornithine yield normalized to DCW are shown.

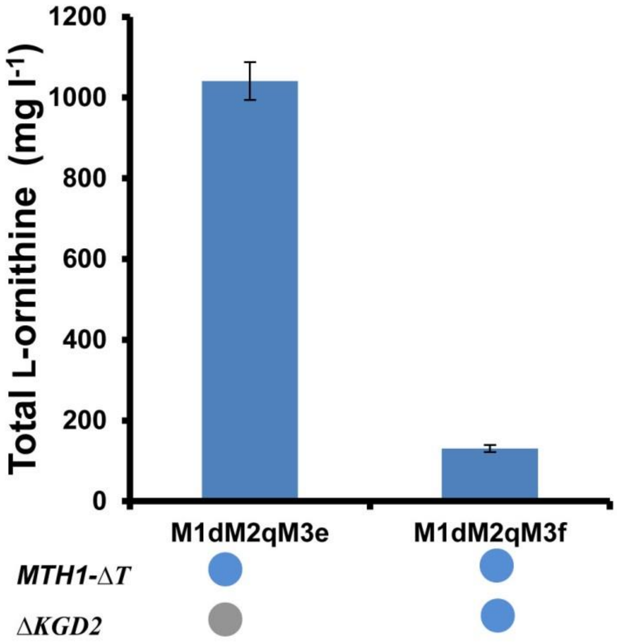

Supplementary Figure 16| Deletion of *KGD2* decreased the L-ornithine titre. All strains were cultivated for 72 h in minimal media. All data are presented as the mean ± s.d. (n≥3).

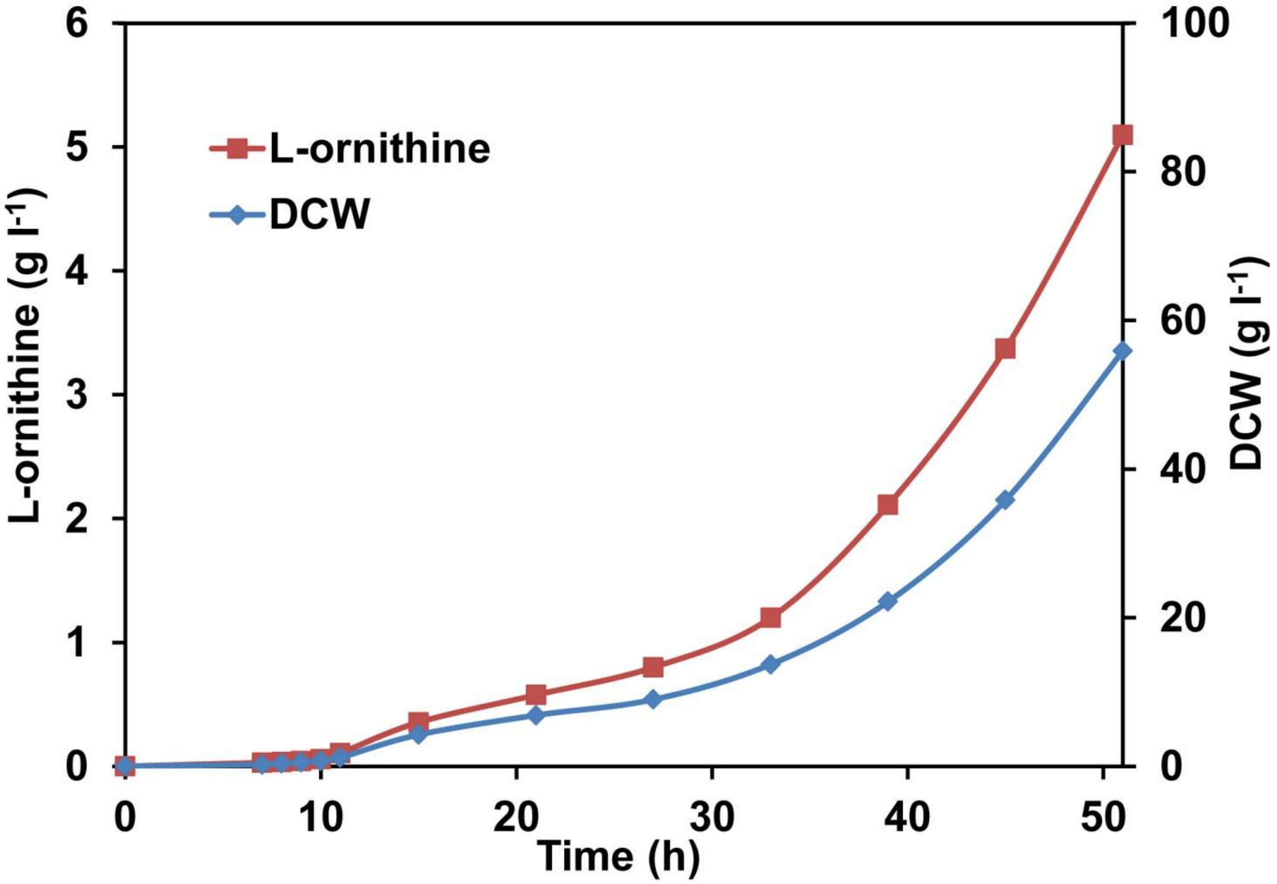

Supplementary Figure 17| Fed-batch fermentation of the engineered strain in a 3-l bioreactor. Time course profile of dry cell weight (DCW) and L-ornithine titre of strain M1cM2qM3a. For fed-batch cultivation, the strain was first grown in a pre-culture with Delft medium. A 3-l bioreactor with an initial volume of 900 ml Delft medium was inoculated at a cell density of 0.05. Cells were cultivated at 30 ºC, 600 rpm agitation, 1 vvm air flow, dissolved oxygen above 30% and pH 5.5. After the glucose and part of the ethanol were consumed, the exponential feed was started.

Supplementary Table 1: Background strains constructed in this study

No	Strain name	Genotype	Genus/Species	Reference
**1**	ORN-A	*MATa SUC2 MAL2-8c ura3-52 his3-Δ1* P_{ARG3}::P_{HXT1}	*Saccharomyces cerevisiae*	This study
**2**	ORN-B	*MATa SUC2 MAL2-8c ura3-52 his3-Δ1* P_{ARG3}::P_{KEX2}	*Saccharomyces cerevisiae*	This study
**3**	ORN-E(KanMX)	*MATa SUC2 MAL2-8c ura3-52 his3-Δ1* P_{ARG3}::P_{KEX2} *car2Δ::LoxP-KanMX-LoxP*	*Saccharomyces cerevisiae*	This study
**4**	ORN-F(KanMX)	*MATa SUC2 MAL2-8c ura3-52 his3-Δ1* P_{ARG3}::P_{KEX2} *carΔ2::LoxP-KanMX-LoxP-*P_{TPI}-*ORT1*-T_{pYX212}	*Saccharomyces cerevisiae*	This study
**5**	ORN-G(KanMX)	*MATa SUC2 MAL2-8c ura3-52 his3-Δ1* P_{ARG3}::P_{KEX2} *car2Δ::LoxP-KanMX-LoxP-*T_{CTC1}-*AGC1*-P_{tHXT1}-P_{TPI}-*ORT1*-T_{pYX212}	*Saccharomyces cerevisiae*	This study
**6**	ORN-F	*MATa SUC2 MAL2-8c ura3-52 his3-Δ1* P_{ARG3}::P_{KEX2} *car2Δ::LoxP-*P_{TPI}-*ORT1*-T_{pYX212}	*Saccharomyces cerevisiae*	This study
**7**	ORN-G	*MATa SUC2 MAL2-8c ura3-52 his3-Δ1* P_{ARG3}::P_{KEX2} *car2Δ::LoxP-*T_{CTC1}-*AGC1*-P_{tHXT1}-P_{TPI}-*ORT1*-T_{pYX212}	*Saccharomyces cerevisiae*	This study
**8**	ORN-H(KanMX)	*MATa SUC2 MAL2-8c ura3-52 his3-Δ1* P_{ARG3}::P_{KEX2} *car2Δ::LoxP-*P_{TPI}-*ORT1*-T_{pYX212} *ura3:: LoxP-KanMX-LoxP-*P_{TEF1}-*GDH1*-T_{DIT1}	*Saccharomyces cerevisiae*	This study
**9**	ORN-I(KanMX)	*MATa SUC2 MAL2-8c ura3-52 his3-Δ1* P_{ARG3}::P_{KEX2} *car2Δ::LoxP-*P_{TPI}-*ORT1*-T_{pYX212} *ura3:: LoxP-KanMX-LoxP-*T_{TDH2}-*ODC1*-P_{PGK1}-P_{TEF1}-*GDH1*-T_{DIT1}	*Saccharomyces cerevisiae*	This study
**10**	ORN-J(KanMX)	*MATa SUC2 MAL2-8c ura3-52 his3-Δ1* P_{ARG3}::P_{KEX2} *car2Δ::LoxP-*P_{TPI}-*ORT1*-T_{pYX212} *ura3:: LoxP-KanMX-LoxP-*P_{TEF1}-*MLS-GDH1*-T_{DIT1}	*Saccharomyces cerevisiae*	This study
**11**	ORN-K(KanMX)	*MATa SUC2 MAL2-8c ura3-52 his3-Δ1* P_{ARG3}::P_{KEX2} *car2Δ::LoxP-*P_{TPI}-*ORT1*-T_{pYX212} *ura3:: LoxP-KanMX-LoxP-*P_{TEF1}-*MLS-GDH2*-T_{DIT1}	*Saccharomyces cerevisiae*	This study
**12**	ORN-J	*MATa SUC2 MAL2-8c ura3-52 his3-Δ1* P_{ARG3}::P_{KEX2} *car2Δ::LoxP-PTPI-ORT1-*T_{pYX212} *ura3:: LoxP-*P_{TEF1}-*MLS-GDH1*-T_{DIT1}	*Saccharomyces cerevisiae*	This study
**13**	ORN-L(KanMX)	*MATa SUC2 MAL2-8c ura3-52 his3-Δ1* P_{ARG3}::P_{KEX2} *car2Δ::LoxP-*T_{CTC1}-*AGC1*-P_{tHXT1}-P_{TPI}-*ORT1*-T_{pYX212} *ura3:: LoxP-KanMX-LoxP-*P_{TEF1}-*GDH1*-T_{DIT1}	*Saccharomyces cerevisiae*	This study
**14**	ORN-M(KanMX)	*MATa SUC2 MAL2-8c ura3-52 his3-Δ1* P_{ARG3}::P_{KEX2} *car2Δ::LoxP-*T_{CTC1}-*AGC1*-P_{tHXT1}-P_{TPI}-*ORT1*-T_{pYX212} *ura3:: LoxP-KanMX-LoxP-*P_{TEF1}-*GDH3*-T_{DIT1}	*Saccharomyces cerevisiae*	This study
**15**	ORN-N(KanMX)	*MATa SUC2 MAL2-8c ura3-52 his3-Δ1* P_{ARG3}::P_{KEX2} *car2Δ::LoxP-*T_{CTC1}-*AGC1*-P_{tHXT1}-P_{TPI}-*ORT1*-T_{pYX212}*ura3:: LoxP-KanMX-LoxP-*T_{TDH2}-*ODC1*-P_{PGK1}-P_{TEF1}-*GDH3*-T_{DIT1}	*Saccharomyces cerevisiae*	This study

**16**	ORN-O(KanMX)	*MATa SUC2 MAL2-8c ura3-52 his3-Δ1* P_{ARG3}::P_{KEX2} *car2Δ::LoxP*-T_{CTC1}-*AGC1*-P_{tHXT1}-P_{TPI}-*ORT1*-T_{pYX212}*ura3:: LoxP-KanMX-LoxP*-T_{TDH2}-*ODC1*-P_{PGK1}-P_{TEF1}-*GDH1*-T_{DIT1}	*Saccharomyces cerevisiae*	This study
**17**	ORN-P(KanMX)	*MATa SUC2 MAL2-8c ura3-52 his3-Δ1* P_{ARG3}::P_{KEX2} *car2Δ::LoxP*-T_{CTC1}-*AGC1*-P_{tHXT1}-P_{TPI}-*ORT1*-T_{pYX212} *YPRτ3:: LoxP-KanMX-LoxP*-P_{TEF1}-*AOX1*-T_{PRM9}	*Saccharomyces cerevisiae*	This study
**18**	ORN-Q(KanMX)	*MATa SUC2 MAL2-8c ura3-52 his3-Δ1* P_{ARG3}::P_{KEX2} *car2Δ::LoxP*-T_{CTC1}-*AGC1*-P_{tHXT1}-P_{TPI}-*ORT1*-T_{pYX212}*YPRτ3:: LoxP-KanMX-LoxP*-T_{pYX212}-*NDI1*-P_{PGK1}-P_{TEF}*1-AOX1*-T_{PRM9}	*Saccharomyces cerevisiae*	This study
**19**	ORN-R(KanMX)	*MATa SUC2 MAL2-8c ura3-52 his3-Δ1* P_{ARG3}::P_{KEX2} *car2Δ::LoxP*-T_{CTC1}-*AGC1*-P_{tHXT1}-P_{TPI}-*ORT1*-T_{pYX212} *ura3:: LoxP-KanMX-LoxP*-P_{TEF1}-*MLS-GDH1*-T_{DIT1}	*Saccharomyces cerevisiae*	This study
**20**	ORN-L	*MATa SUC2 MAL2-8c ura3-52 his3-Δ1* P_{ARG3}::P_{KEX2} *car2Δ::LoxP*-T_{CTC1}-*AGC1*-P_{tHXT1}-P_{TPI}-*ORT1*-T_{pYX212} *ura3:: LoxP*-P_{TEF1}-*GDH1*-T_{DIT1}	*Saccharomyces cerevisiae*	This study
**21**	ORN-O	*MATa SUC2 MAL2-8c ura3-52 his3-Δ1* P_{ARG3}::P_{KEX2} *car2Δ::LoxP*-T_{CTC1}-*AGC1*-P_{tHXT1}-P_{TPI}-*ORT1*-T_{pYX212}*ura3:: LoxP*-T_{TDH2}-*ODC1*-P_{PGK1}-P_{TEF1}-*GDH1*-T_{DIT1}	*Saccharomyces cerevisiae*	This study
**22**	ORN-S(KanMX)	*MATa SUC2 MAL2-8c ura3-52 his3-Δ1* P_{ARG3}::P_{KEX2} *car2Δ::LoxP*-T_{CTC1}-*AGC1*-P_{tHXT1}-P_{TPI}-*ORT1*-T_{pYX212} *ura3:: LoxP-PTEF1-GDH1*-T_{DIT1} *kgd2Δ:: LoxP-KanMX-LoxP*	*Saccharomyces cerevisiae*	This study
**23**	ORN-T(KanMX)	*MATa SUC2 MAL2-8c ura3-52 his3-Δ1* P_{ARG3}::P_{KEX2} *car2Δ::LoxP*-T_{CTC1}-*AGC1*-P_{tHXT1}-P_{TPI}-*ORT1*-T_{pYX212} *ura3:: LoxP*-P_{TEF1}-*GDH1*-T_{DIT1} *kgd2Δ:: LoxP-KanMX-LoxP*-T_{PRM9t}-*MTH1*-P_{TEF1}	*Saccharomyces cerevisiae*	This study
**24**	ORN-U(KanMX)	*MATa SUC2 MAL2-8c ura3-52 his3-Δ1* P_{ARG3}::P_{KEX2} *car2Δ::LoxP*-T_{CTC1}-*AGC1*-P_{tHXT1}-P_{TPI}-*ORT1*-T_{pYX212} *ura3:: LoxP*-P_{TEF1}-*GDH1*-T_{DIT1} *kgd2Δ:: LoxP-KanMX-LoxP*-T_{PRM9t}-*AOX1*-P_{TEF1}	*Saccharomyces cerevisiae*	This study
**25**	ORN-V(KanMX)	*MATa SUC2 MAL2-8c ura3-52 his3-Δ1* P_{ARG3}::P_{KEX2} *car2Δ::LoxP*-T_{CTC1}-*AGC1*-P_{tHXT1}-P_{TPI}-*ORT1*-T_{pYX212} *ura3:: LoxP*-P_{TEF1}-*GDH1*-T_{DIT1} *YPRτ3:: LoxP-KanMX-LoxP*-P_{TEF1}-*MTH1*-T_{PRM9}	*Saccharomyces cerevisiae*	This study

**26**	ORN-WN(KanMX)	*MATa SUC2 MAL2-8c ura3-52 his3-Δ1 P_{ARG3}::P_{KEX2} car2Δ::LoxP-T_{CTC1}-AGC1-P_{tHXT1}-P_{TPI}-ORT1-T_{pYX212} ura3:: LoxP-P_{TEF1}-GDH1-T_{DIT1} YPRτ3:: LoxP-KanMX-LoxP- T_{pYX212}-NDI1-P_{PGK}-P_{TEF1}-AOX1-T_{PRM9}*	*Saccharomyces cerevisiae*	This study
**27**	ORN-W(KanMX)	*MATa SUC2 MAL2-8c ura3-52 his3-Δ1 P_{ARG3}::P_{KEX2} car2Δ::LoxP-T_{CTC1}-AGC1-P_{tHXT1}-P_{TPI}-ORT1-T_{pYX212} ura3:: LoxP-P_{TEF1}-GDH1-T_{DIT1} YPRτ3:: LoxP-KanMX-LoxP-P_{TEF1}-AOX1-T_{PRM9}*	*Saccharomyces cerevisiae*	This study
**28**	ORN-X(KanMX)	*MATa SUC2 MAL2-8c ura3-52 his3-Δ1 P_{ARG3}::P_{KEX2} car2Δ::LoxP-T_{CTC1}-AGC1-P_{tHXT1}-P_{TPI}-ORT1-T_{pYX212}ura3:: LoxP-T_{TDH2}-ODC1-P_{PGK1}-P_{TEF1}-GDH1-T_{DIT1} YPRτ3:: LoxP-KanMX-LoxP-P_{TEF1}-AOX1-T_{PRM9}*	*Saccharomyces cerevisiae*	This study
**29**	ORN-Y(KanMX)	*MATa SUC2 MAL2-8c ura3-52 his3-Δ1 PARG3::PKEX2 car2Δ::LoxP-TCTC1-AGC1-PtHXT1-PTPI-ORT1-TpYX212ura3:: LoxP-T_{TDH2}-ODC1-P_{PGK1}-P_{TEF1}-GDH1-T_{DIT1} kgd2:: LoxP-KanMX-LoxP-T_{PRM9t}-AOX1-P_{TEF1}*	*Saccharomyces cerevisiae*	This study
**30**	ORN-Z(KanMX)	*MATa SUC2 MAL2-8c ura3-52 his3-Δ1 P_{ARG3}::P_{KEX2} car2Δ::LoxP-T_{CTC1}-AGC1-P_{tHXT1}-P_{TPI}-ORT1-T_{pYX212}ura3:: LoxP-T_{TDH2}-ODC1-P_{PGK1}-P_{TEF1}-GDH1-T_{DIT1} kgd2:: LoxP-KanMX-LoxP*	*Saccharomyces cerevisiae*	This study
**31**	B0166A CEN.PK	*MATa ort1Δ*	*Saccharomyces cerevisiae*	Euroscarf

Supplementary Table 2: Strains used for module and full pathway optimization

No	Strain name	Module 1		Module 2		Module 3		Reference		
		Module	Description	Module	Description	Module	Description	Background strain	Plasmid 1(URA)	Plasmid 2(HIS)
1	M1a	M1a	P_{HXT1}-*ARG3*	Null	Null	Null	Null	ORN-A	pYX212	p423GPD
2	M1b	M1b	P_{KEX2}-*ARG3*	Null	Null	Null	Null	ORN-B	pYX212	p423GPD
3	M1c	M1c	P_{KEX2}-*ARG3; CAR2Δ*	Null	Null	Null	Null	ORN-E(KanMX)	pYX212	p423GPD
4	M1bM2f	M1b	P_{KEX2}-*ARG3*	Null	Null	Null	Null	ORN-B	pYX212	GO1
5	M1bM2g	M1b	P_{KEX2}-*ARG3*	Null	Null	Null	Null	ORN-B	pYX212	GO2
6	M1bM2r	M1b	P_{KEX2}-*ARG3*	Null	Null	Null	Null	ORN-B	pYX212	P_{ADH1}-tGCN4
7	M1cM2a	M1c	P_{KEX2}-*ARG3; car2Δ*	M2a	*ARG2*	Null	Null	ORN-E(KanMX)	pSPGM1-ARG2	p423GPD
8	M1cM2b	M1c	P_{KEX2}-*ARG3; car 2Δ*	M2b	*ARG5,6*	Null	Null	ORN-E(KanMX)	pSPGM1-ARG5,6	p423GPD
9	M1cM2c	M1c	P_{KEX2}-*ARG3; car 2Δ*	M2c	*ARG7*	Null	Null	ORN-E(KanMX)	pSPGM1-ARG7	p423GPD
10	M1cM2d	M1c	P_{KEX2}-*ARG3; car 2Δ*	M2d	*MLS-argB*$_{Cg}$	Null	Null	ORN-E(KanMX)	pYX212-argB$_{Ec}$	p423GPD
11	M1cM2e	M1c	P_{KEX2}-*ARG3; car 2Δ*	M2e	*MLS-argJ*$_{Cg}$	Null	Null	ORN-E(KanMX)	pYX212-argJ$_{Cg}$	p423GPD
12	M1cM2f	M1c	P_{KEX2}-*ARG3; car 2Δ*	M2f	*ARG5,6; ARG7; ARG8*	Null	Null	ORN-E(KanMX)	pYX212	GO1
13	M1cM2g	M1c	P_{KEX2}-*ARG3; car 2Δ*	M2g	*ARG5,6; ARG7; ARG8; ARG2*	Null	Null	ORN-E(KanMX)	pYX212	GO2
14	M1cM2h	M1c	P_{KEX2}-*ARG3; car 2Δ*	M2h	*ARG5,6; ARG7; ARG8; ARG2; ORT1*	Null	Null	ORN-F	pYX212	GO2

15	M1cM2i	M1c	P_{KEX2}-*ARG3; car 2Δ*	M2i	*ARG5,6; ARG7; ARG8; ARG2; ORT1; MLS-GDH1*	Null	Null	ORN-J(KanMX)	pYX212	GO2
16	M1cM2j	M1c	P_{KEX2}-*ARG3; car 2Δ*	M2j	*ARG5,6; ARG7; ARG8; ARG2; ORT1; MLS-GDH2*	Null	Null	ORN-K(KanMX)	pYX212	GO2
17	M1cM2k	M1c	P_{KEX2}-*ARG3; car 2Δ*	M2k	*ARG5,6; ARG7; ARG8; ARG2; ORT1; AGC1*	Null	Null	ORN-G	pYX212	GO2
18	M1cM2l	M1c	P_{KEX2}-*ARG3; car 2Δ*	M2l	*ARG5,6; ARG7; ARG8; ARG2; ORT1; AGC1; GDH1*	Null	Null	ORN-L(KanMX)	pYX212	GO2
19	M1cM2m	M1c	P_{KEX2}-*ARG3; car 2Δ*	M2m	*ARG5,6; ARG7; ARG8; ARG2; ORT1; AGC1; GDH3*	Null	Null	ORN-M(KanMX)	pYX212	GO2
20	M1cM2n	M1c	P_{KEX2}-*ARG3; car 2Δ*	M2n	*ARG5,6; ARG7; ARG8; ARG2; ORT1; AGC1; GLN1; GLT1*	Null	Null	ORN-G	YO1	GO2
21	M1cM2o	M1c	P_{KEX2}-*ARG3; car 2Δ*	M2o	*ARG5,6; ARG7; ARG8; ARG2; ORT1; AGC1; GDH3; ODC1*	Null	Null	ORN-N(KanMX)	pYX212	GO2
22	M1cM2p	M1c	P_{KEX2}-*ARG3; car 2Δ*	M2p	*ARG5,6; ARG7; ARG8; ARG2; ORT1; AGC1; GDH1; ODC1*	Null	Null	ORN-O(KanMX)	pYX212	GO4
23	M1cM2q	M1c	P_{KEX2}-*ARG3; car 2Δ*	M2q	$argA_{Ec;}$ $argB_{Ec}$; $argC_{Cg}$; $argD_{Cg}$; $argJ_{Cg}$; *ORT1; AGC1; GDH1*	Null	Null	ORN-O(KanMX)	pYX212	GO4
24	M1cM2r	M1c	P_{KEX2}-*ARG3;*	M2r	*PADH1- tGCN4*	Null	Null	ORN-	pYX212	P_{ADH1}-

			CAR2Δ					E(KanMX)		tGCN4
25	M1cM2s	M1c	P_{KEX2}-*ARG3; car 2Δ*	M2s	*PTEF1- tGCN4*	Null	Null	ORN-E(KanMX)	pYX212	P_{TEF1}-tGCN4
26	M1cM2t	M1c	P_{KEX2}-*ARG3; car 2Δ*	M2t	*PGPD1- tGCN4*	Null	Null	ORN-E(KanMX)	pYX212	P_{GPD1}-tGCN4
27	M1cM2qM3a	M1c	P_{KEX2}-*ARG3; car 2Δ*	M2q	$argA_{Ec;}$ $argB_{Ec}$; $argC_{Cg}$; $argD_{Cg}$; $argJ_{Cg}$; *ORT1; AGC1; GDH1*	M3a	*PDA1;CIT1;ACO2;IDP1;PYC2*	ORN-L(KanMX)	YO4	GO4
28	M1cM2qM3b	M1c	P_{KEX2}-*ARG3; car 2Δ*	M2q	$argA_{Ec;}$ $argB_{Ec}$; $argC_{Cg}$; $argD_{Cg}$; $argJ_{Cg}$; *ORT1; AGC1; GDH1*	M3b	*PDA1[S313A];CIT1;ACO2;IDP1;PYC2*	ORN-L(KanMX)	YO5	GO4
29	M1cM2qM3c	M1c	P_{KEX2}-*ARG3; car 2Δ*	M2q	$argA_{Ec;}$ $argB_{Ec}$; $argC_{Cg}$; $argD_{Cg}$; $argJ_{Cg}$; *ORT1; AGC1; GDH1*	M3c	*HaAOX1*	ORN-W(KanMX)	pYX212	GO4
30	M1cM2qM3d	M1c	P_{KEX2}-*ARG3; car 2Δ*	M2q	$argA_{Ec;}$ $argB_{Ec}$; $argC_{Cg}$; $argD_{Cg}$; $argJ_{Cg}$; *ORT1; AGC1; GDH1*	M3d	*HaAOX1; NDI1*	ORN-WN(KanMX)	pYX212	GO4
31	M1cM2qM3e	M1c	P_{KEX2}-*ARG3; car 2Δ*	M2q	$argA_{Ec;}$ $argB_{Ec}$; $argC_{Cg}$; $argD_{Cg}$; $argJ_{Cg}$; *ORT1; AGC1; GDH1*	M3e	*MTH1-ΔT*	ORN-V(KanMX)	pYX212	GO4
32	M1cM2qM3f	M1c	P_{KEX2}-*ARG3; car 2Δ*	M2q	$argA_{Ec;}$ $argB_{Ec}$; $argC_{Cg}$; $argD_{Cg}$; $argJ_{Cg}$; *ORT1; AGC1; GDH1*	M3f	*MTH1-ΔT; kgd2Δ*	ORN-T(KanMX)	pYX212	GO4
33	M1dM2q	M1d	P_{KEX2}-*ARG3; car 2Δ; CAR1*	M2q	$argA_{Ec;}$ $argB_{Ec}$; $argC_{Cg}$; $argD_{Cg}$; $argJ_{Cg}$; *ORT1; AGC1; GDH1*	Null	Null	ORN-L(KanMX)	TPIp-CAR1	GO4

34	M1dM2qM3c	M1d	*P_{KEX2}-ARG3; car 2Δ; CAR1*	M2q	*$argA_{Ec}$; $argB_{Ec}$; $argC_{Cg}$; $argD_{Cg}$; $argJ_{Cg}$; ORT1; AGC1; GDH1*	M3c	*HaAOX1*			
35	M1dM2qM3e	M1d	*P_{KEX2}-ARG3; car 2Δ; CAR1*	M2q	*$argA_{Ec}$; $argB_{Ec}$; $argC_{Cg}$; $argD_{Cg}$; $argJ_{Cg}$; ORT1; AGC1; GDH1*	M3e	*MTH1-ΔT*	ORN-V(KanMX)	TPIp-CAR1	GO4
36	M1dM2qM3f	M1d	*P_{KEX2}-ARG3; car 2Δ; CAR1*	M2q	*$argA_{Ec}$; $argB_{Ec}$; $argC_{Cg}$; $argD_{Cg}$; $argJ_{Cg}$; ORT1; AGC1; GDH1*	M3f	*MTH1-ΔT; kgd2Δ*	ORN-T(KanMX)	TPIp-CAR1	GO4
37	B0166A(ORT1)	Null	Null	Null	Null	Null	Null	B0166A CEN.PK	pYX212	GO4

Supplementary Table 3: Plasmids used in this study

No	Plasmid/Alias Name	Genes Inserted	Backbone Vector	Reference
1	GO1	*ARG5,6; ARG7; ARG8*	p423GPD	This Study
2	GO2	*ARG5,6 ; ARG7; ARG8; ARG2*	p423GPD	This Study
3	GO3	*ARG5,6 ARG7; ARG8; ARG2; CAR1*	p423GPD	This Study
4	GO4	$argB_{Ec;}$ $argA_{E;\ c}$ $argC_{Cg;}$ $argD_{Cg}$; $argJ_{Cg}$	p423GPD	This Study
5	YO1	*GLN1; GLT1*	pYX212	This Study
6	YO2	*PYC2;CIT1; IDP1*	pYX212	This Study
7	YO3	*GLN1;GLT1; PYC2; CIT1; IDP1*	pYX212	This Study
8	YO4	*PDA1; ACO2 ;PYC2; CIT1; IDP1*	pYX212	This Study
9	YO5	*mPDA1;ACO2; PYC2; CIT1; IDP1*	pYX212	This Study
10	pSPGM1-ARG2	*ARG2*	pSPGM1	This Study
11	pSPGM1-ARG5,6	*ARG5,6*	pSPGM1	This Study
12	pSPGM1-ARG7	*ARG7*	pSPGM1	This Study
13	pYX212-MLSargB$_{Cg}$	$MLS\text{-}argB_{Cg}$	pYX212	This Study
14	pYX212-MLS-argJ$_{Cg}$	$MLS\text{-}argJ_{Cg}$	pYX212	This Study
15	PADH1- tGCN4	*tGCN4*	p423ADH	This Study
16	PTEF1- tGCN4	*tGCN4*	p423TEF	This Study
17	PGPD1- tGCN4	*tGCN4*	p423GPD	This Study
18	TPIp-CAR1	*CAR1*	pYX212	[1]
19	pRS416-PDA1 [S313A]	*PDA1 [S313A]*	pRS416	[2]
20	pRS416-PDA1	*PDA1*	pRS416	[2]

Supplementary Table 4: Primers used in this study

No	Primer Name	Sequence (5' to 3')
1	TDH3p-F(YJ)	TCGAGTTTATCATTATCAATACTGCCATTTCAAAGAATAC
2	TDH3p-R(YJ)	GTTTGTTTATGTGTGTTTATTCGAAACTAAGTTCTTGGTG
3	ARG5,6-F1	GAACTTAGTTTCGAATAAACACACATAAACAAACAAAATGCCATCTGCTAGCTTACTC
4	ARG5,6-R1	CTAAATCATTAAAGTAACTTAAGGAGTTAAATTCAGACACCAATAATTTTATTTTC
5	TDH2t-F1(YJ)	ATTTAACTCCTTAAGTTACTTTAATGATTTAGTTTTTA
6	ADH1t-F(YJ)	GCGAATTTCTTATGATTTATGATTTTTATTATTAAATAAG
7	ARG8-R1	CTTATTTAATAATAAAAATCATAAATCATAAGAAATTCGCTTAAGCGTAAACCGCTTC
8	ARG8-F1	GAAGTAATTATCTACTTTTTACAACAAATATAAAACAATGTTTAAAAGATATTTATC
9	PGK1p-R1(YJ)	CATTTTGTTATATTTGTTGTAAAAAGTAGATAATTACTTCC
10	TEF1p-R1(YJ)	CATTTTGTAATTAAAACTTAGATTAGATTGCTATGCTTTC
11	ARG7-F1	GAAAGCATAGCAATCTAATCTAAGTTTTAATTACAAAATGAGAATATCATCAACATTG
12	ARG7-R1	CTAATTACATGACTCGAGGTCGACGGTATCTTATGAACGGTAATCACCGTTAATTG
13	CYC1t-F(YJ)	GATACCGTCGACCTCGAGTCATGTAATTAGTTATGTC
14	CYC1t-R(YJ)	GGGTACCGGCCGCAAATTAAAGCCTTCGAGCGTCCC
15	ARG7-R2	CATTAAAAAACTATATCAATTAATTTGAATTAACTTATGAACGGTAATCACCGTTAATTG
16	FBA1t-F2(YJ)	GTTAATTCAAATTAATTGATATAGTTTTTTAATGAG
17	FBA1t-R3	GAAGATGTTCTTATCCAAATTTCAACTGTTATATAAGTAAGCTACTATGAAAGAC
18	TPI1t-R1	TATATAACAGTTGAAATTTGGATAAGAACATC
19	TPI1t-F1	GATTAATATAATTATATAAAAATATTATCTTC

20	ARG2-R1	GATAATATTTTTATATAATTATATTAATCTCATGAAATATTTTTTTCATTTTCCCAAC
21	ARG2-F1	CACAAAAACAAAAAGTTTTTTTAATTTTAATCAAAAAATGTGGAGGAGAATATTCGC
22	tHXT7p-R1	GATTAAAATTAAAAAAACTTTTTGTTTTTGTG
23	tHXT7p-F1	CTCAAAAAAATGGCATTATTCTAAGTAAGTTAAATATCCCTCGTAGGAACAATTTCG
24	TPIp-F1(YJ)	GGATATTTAACTTACTTAGAATAATG
25	TPIp-R1	CTAATTACATGACTCGAGGTCGACGGTATCTTACATTTTTAGTTTATGTATGTG
26	TPIp-F1(YJ)	GTTTAAAGAGATTACGGATATTTAACTTACTTAGAATAATG
27	TPIp-R2	CATTTTTAGTTTATGTATGTGTTTTTTGTAGTTATAG
28	GLN1-F1	CTATAACTACAAAAAACACATACATAAACTAAAAATGGCTGAAGCAAGCATCG
29	GLN1-R1	CTCATTAAAAAACTATATCAATTAATTTGAATTAACTTATGAAGATTCTCTTTC
30	FBA1t-F2(YJ)	GTTAATTCAAATTAATTGATATAGTTTTTTAATGAG
31	CYC1t-F(YJ)	GATACCGTCGACCTCGAGTCATGTAATTAGTTATGTC
32	GLT1B-R1	CATAACTAATTACATGACTCGAGGTCGACGGTATCTTAGACTTGACTAGCTAATTC
33	GLT1B-F1	CTCAGGGTGCTAAGCCGGGTGAAGGTGGTGAACTAC
34	GLT1A-R1	CTCAATTGACCATCGGTTTGGACAACAACATTACGTC
35	GLT1A-F1	GTAATTATCTACTTTTTACAACAAATATAACAAAATGCCAGTGTTGAAATCAGAC
36	PGK1p-R1(YJ)	CATTTTGTTATATTTGTTGTAAAAAGTAGATAATTACTTCC
37	TEF1p-R2	GATACCCGGGTCGACGCGTAAGCTTGTGGGCCCTATCATTTGTAATTAAAACTTAG
38	TPIp-R3	CTCATTAAAAAACTATATCAATTAATTTGAATTAACTTTTAGTTTATGTATGTG
39	PGK1p-R2	CATAACTAATTACATGACTCGAGGTCGACGGTATCCATTTTGTTATATTTGTTG
40	PYC2-F1	GAAAGCATAGCAATCTAATCTAAGTTTTAATTACAAAATGAGCAGTAGCAAGAAATTG

41	PYC2-R1	CTAAATCATTAAAGTAACTTAAGGAGTTAAATTTACTTTTTTTGGGATGGG GGTAG
42	TDH2t-F1(YJ)	ATTTAACTCCTTAAGTTACTTTAATGATTTAGTTTTTA
43	ADH1t-F(YJ)	GCGAATTTCTTATGATTTATGATTTTTATTATTAAATAAG
44	CIT1-R1	CTTATTTAATAATAAAAATCATAAATCATAAGAAATTCGCTTAGTTCTTAC TTTCGATTTTC
45	CIT1-F1	GAACTTAGTTTCGAATAAACACACATAAACAAACAAAATGTCAGCGATAT TATCAAC
46	TDH3p-R1	CATTTTGTTTGTTTATGTGTGTTTATTCGAAACTAAG
47	tHXT7p-F(YJ)	GTATTCTTTGAAATGGCAGTATTGATAATGATAAACTCGAGCTCGTAGGAA CAATTTCG
48	tHXT7p-R1	GATTAAAATTAAAAAAACTTTTTGTTTTTGTG
49	IDP1-F1	CACAAAAACAAAAAGTTTTTTTAATTTTAATCAAAAAATGAGTATGTTATC TAGAAG
50	IDP1-R1	GATACCCGGGTCGACGCGTAAGCTTGTGGGCCCTATTACTCGATCGACTTG ATTTC
51	Pyx212t-F(YJ)	TAGGGCCCACAAGCTTACGCGTCGACCCGGGTATCC
52	Pyx212t-R(YJ)	TGCCGTAAACCACTAAATCGGAACCCTAAAGG
53	ARG3p-L-F	CACAAAGGTTATCCCTATTTGAAAACAGAGATAGC
54	AR3p-L-R	CATGTCGAGATACGTGTATCTACATCAGAGCTGCTGACGATACTGCTGTGA GTTGCAC
55	KiURA3-F	CTCGTCACATAATTATAAACTACTAACCCATTATCAGATGGTGGTTATTCG TGGATC
56	KiURA3-R	GTCGAGATACGTGTATCTACATCAGAGCTGCTGATGATGTAGTTTCTGGTT TTTAAATC
57	KEX2p-F	TCAGCAGCTCTGATGTAG
58	KEX2p-R	CTGATAATGGGTTAGTAG
59	ARG3-L-F	CTCGTCACATAATTATAAACTACTAACCCATTATCAGATGTCAACCACAGC ATCCAC
60	ARG3-L-R	GAGAGTTGATGATCGGTACAGAGGAATCCTTG
61	KGD2p-L-F	CTGTTGTTGTGGGACATATTCGAACGTCTCGTGGAAAG
62	KGD2p-L-R	CATGTCGAGATACGTGTATCTACATCAGAGCTGCTGAGCTGATAGACCAA

		GTGTGGATC
63	KGD2-L-F	CTCGTCACATAATTATAAACTACTAACCCATTATCAGATGCTTTCCAGAGC GACGCG
64	KGD2-L-R	CAGTGACAGTGTCCTCTGGTTTGAAATTTAGCTTC
65	ARG3p-L-F2	GATGATGAAGAGGATAGTGACAGTGAC
66	KiURA3-R1	CAATCACAACCACATCTTAGATAGTTG
67	KiURA3-F1	GATGCATTGGGTCAGCAGTACAG
68	ARG3-L-R2	CTTGCAAAGGGTGGAATTTGTC
69	KGD2p-L-F2	GTCAATGCTATTTGCATACTGCC
70	KGD2-L-R2	CTCGACCTGAGCTAACTCCTCAC
71	GLN1-F2	CAAGGAAGGTTACGGTTACTTTGAAG
72	GLT1B-R2	GAAAGACTTTTGCATGTGGTGACTG
73	CIT1-F2	GCTACTATAGTGGCGAGCATTCAATAG
74	IDP1-R2	CAGGTTGAATTCCTTCACACGAG
75	ARG5,6-F2	GAATGATGCTGAAGATCGTGTAGTTG
76	ARG8-R2	CCAGAGAATTGGGACTTTTGATC
77	ORT1-F1	CCGGAATTC AATAAAATGGAGGACAGTAAAAAGAAAGG
78	ORT1-R1	CCGCTCGAGTTAAAGTGCAGAAAGAGTCTCAAAG
79	ORT1-R2	CCCGGGGATTCGCATAGAACCACCACCACCAAGTGCAGAAAGAGTCTCAA AGATG
80	AQR1-F1	CCGGAATTC AATAAAATGTCACGAAGTAACAGTATATAC
81	AQR1-R1	CCGCTCGAGTTAATTATGATTATCGTTCTGG
82	AQR1-R2	GTTAATTAACCCGGGGATTCGCATAGAACCACCACCATTATGATTATCGTT CTGG
83	GFP-F1	GGTGGTGGTGGTTCTATGCGAATCCCCGGGTTAATTAAC
84	GFP-R2-PYX212	CCGCTCGAGCTATTTGTATAGTTCATC
85	MLS-F1	CGCGGATCCAATAAAATGTTGTCCTTGAGACAATCCATC
86	MLS-argJ-F2	CGCGGATCCAATAAAATGGCCGAAAAAGGTATAACAG

87	MLS-argJ-R1	CCATCGATTTATGAAGAGTAAGCGGAATTAATTTC
88	MLS-argJ-R2	CCCGGGGATTCGCATAGAACCACCACCACCTGAAGAGTAAGCGGAATTAATTTCG
89	MLS-argB-R1	CCATCGATTTATAATTCACCGTCCTTGTCGTC
90	GFP-R1-P423GPD	CCATCGATCTATTTGTATAGTTCATCC
91	argJ-F3	GAACTTAGTTTCGAATAAACACACATAAACAAACATGGCCGAAAAAGGTATAACAG
92	argJ-R3	CTAAATCATTAAAGTAACTTAAGGAGTTAAATTTATGAAGAGTAAGCGGAATTAATTTC
93	argB-R2	CTTATTTAATAATAAAAATCATAAATCATAAGAAATTCGCTTATAATTCACCGTCCTTGTCG
94	argB-F2	GTAATTATCTACTTTTTACAACAAATATAACAAAATGAATGACTTAATCAAGGACTTG
95	argC-F1	GAAAGCATAGCAATCTAATCTAAGTTTTAATTACAAAATGATAATGCACAATGTCTATGG
96	argC-R1	CATTAAAAAACTATATCAATTAATTTGAATTAACTTAAGGAGCTACACCGACTTGTG
97	argD-R1	GAAGATAATATTTTTATATAATTATATTAATCTTAAGCTATTGTTTCGGCAATTGC
98	argD-F1	CACAAAAACAAAAAGTTTTTTTAATTTTAATCAAAAAATGTCTACATTGGAAACCTGG
99	MLS-F1	GCTTAAATCTATAACTACAAAAAACACATACATAAACTAAAAATGTTGTCCTTGAGACAATCCATC
100	MLS-R1	CTTCTTCGTAAGCTTGTTGAAATTCTGGCTCTGACATTAACAAGTATCTGGAGGAGCATAAAG
101	GDH1-F1	ATGTCAGAGCCAGAATTTCAAC
102	GDH1-F2	GCTTAAATCTATAACTACAAAAAACACATACATAAACTAAAAATGTCAGAGCCAGAATTTCAAC
103	GDH1-R1	CAATACTCATTAAAAAACTATATCAATTAATTTGAATTAACTTAAAATACATCACCTTGGTC
104	ACO1-R1	CATAACTAATTACATGACTCGAGGTCGACGGTATCTTATTTCTTCTCATCGGCCTTAATTTTATTTAAG
105	ACO1-F1	CAAGGAAGTAATTATCTACTTTTTACAACAAATATAACAAAATGCTGTCTGCACGTTCTGC

106	GDH3-F1	GCTTAAATCTATAACTACAAAAAACACATACATAAACTAAAAATGACAAGCGAACCAGAGTTTC
107	GDH3-R1	CAATACTCATTAAAAAACTATATCAATTAATTTGAATTAACTTAAAAAACGTCTCCCTGGTC
108	UPCAR2-F1	GATAAATAAATACCCCACTGGCGG
109	UPCAR2-R1	GTTGTCGACCTGCAGCGTACGAAGCTTCAGGCCTTGATGATGTGAGGATG
110	KanMX-R1	GGCCACTAGTGGATCTGATATCAC
111	CYC1t-R3	GAAGTTATTAGGTGATATCAGATCCACTAGTGGCCGGGTACCGGCCGCAAATTAAAG
112	AGC1-R1	GACATAACTAATTACATGACTCGAGGTCGACGGTATCTCACCCGTTAATGCTTCTTAGG
113	AGC1-F1	CACAAAAACAAAAAGTTTTTTTAATTTTAATCATGGAGCAAATCAATTCGAACAG
114	DOWN-CAR2-F1	GCTCCCTTTAGGGTTCCGATTTAGTGGTTTACGGCACGGCCTCTTGGCTAAGCCCAC
115	DOWN-CAR2-R1	GCAACCAATACAACAGGGGAG
116	TPIp-F3	GAAGTTATTAGGTGATATCAGATCCACTAGTGGCCGGATATTTAACTTACTTAGAATAATG
117	DOWN-CAR2-F2	GAAGTTATTAGGTGATATCAGATCCACTAGTGGCCCGGCCTCTTGGCTAAGCCCAC
118	Up-URA3-F1	GACGTTGAAATTGAGGCTACTGCG
119	Up-URA3-R(amdSYM)	GTTGTCGACCTGCAGCGTACGAAGCTTCAGCTAGGATGAGTAGCAGCACGTTCC
120	Up-Up-UAR3-F1(amdSYM)	GTTATTAGGTGATATCAGATCCACTAGTGGCCGACAAATGAGAACTTCATGTGGG
121	Up-Up-UAR3-R1(TEF)	GGAAGAGTAAAAAAGGAGTAGAAACATTTTGAAGCTATGTTTGACTCTGGTGTTTGTTCATG
122	Up-Up-UAR3-R2(CPS1t)	CATAAATAAAAAAAAAAAGAAGTGTCAAATCAAGTGTCAAATGTTTGACTCTGGTGTTTGTTCATG
123	TEFp-F1	ATAGCTTCAAAATGTTTCTACTCCTTTTTTAC

124	GDH1-F1(TEF)	GAAAGCATAGCAATCTAATCTAAGTTTTAATTACAAAATGTCAGAGCCAGAATTTCAACAAG
125	GDH1-R1(DIT1t)	GGTAGACCAATGTAGCGCTCTTACTTTATTAAAATACATCACCTTGGTCAAAC
126	MLS-GDH1-F1(TEF)	ATGTTATCCTTGAGACAATCCATCAGATTTTTCAAACCTGCTACCAGAACCTTATGCTCTTCCAGATACTTGTTAATGTCAGAGCCAGAATTTCAACAAG
127	GDH3-F1(TEF)	GAAAGCATAGCAATCTAATCTAAGTTTTAATTACAAAATGACAAGCGAACCAGAGTTTCAG
128	GDH3-R1(DIT1t)	GGTAGACCAATGTAGCGCTCTTACTTTACTAAAAAACGTCTCCCTGGTCAAGC
129	MLS-GDH2-F1(TEF)	ATGTTATCCTTGAGACAATCCATCAGATTTTTCAAACCTGCTACCAGAACCTTATGCTCTTCCAGATACTTGTTAATGCTTTTTGATAACAAAAATCGC
130	GDH2-R1(DIT1t)	GGTAGACCAATGTAGCGCTCTTACTTTATCAAGCACTTGCCTCCGCTTCTC
131	GDH2-R2(Tag)	CCATGGATCCACCTCCACCACCTCCAGCACTTGCCTCCGCTTCTC
132	Tag-F1	GGAGGTGGTGGAGGTGGATCCATG
133	Tag-R1(DIT1t)	GGTAGACCAATGTAGCGCTCTTACTTTACTAGGTTGACTTCCCCGCGGAATTC
134	ODC1-F1(PGK)	GTAATTATCTACTTTTTACAACAAATATAACAAAATGACATCTATAGATAATAGACC
135	ODC1-R1(CPS1t)	CTTTGACTATTCAATCATTGCGCTCATTGTTTTTACCATACTTGACTTC
136	CPS1t-F1	GCGCAATGATTGAATAGTCAAAG
137	CPS1t-R1	ATTTGACACTTGATTTGACACTTC
138	DIT1t-F1	TAAAGTAAGAGCGCTACATTGGTC
139	DIT1t-R1	GTTACTCCGCAACGCTTTTCTGAAC
140	Down-URA3-F1(DIT1t)	GTTCAGAAAAGCGTTGCGGAGTAACCATATTTGAGAAGATGCGGCCAGC
141	Down-URA3-R1	CTACACGTTCGCTATGCTTCAAG
142	Diagnostic-F1(URA3)	GTTACAGCAATGAAAGAGCAGAGC
143	Diagnostic-R1(URA3)	CCAAGCCTTGTCCCAAGGCAGCG

144	TEF-R(MLS)	CTGATGGATTGTCTCAAGGATAACATTTTGTAATTAAAACTTAGATTAGATTGCTATGCTTTC
145	Up-YPRCt3-F1	GAGGTGCACGCATTATGGAGACCAC
146	Up-YPRCt3-R1(KanMX)	GTTGTCGACCTGCAGCGTACGAAGCTTCAGCAGGATAGTGGAACACATTCCAAGG
147	KanMX-R2(TEF)	GTAAAAAAGGAGTAGAAACATTTTGAAGCTATGGCCACTAGTGGATCTGATATCAC
148	KanMX-R3(pYX212t)	GGGTTCCGATTTAGTGGTTTACGGCAGGCCACTAGTGGATCTGATATCAC
149	AOX1-F1(TEF)	GAAAGCATAGCAATCTAATCTAAGTTTTAATTACAAAATGTATCCTACTTCTGGTTGTGCTAG
150	AOX1-R1(PRM9t)	GTGCTAGTGTCTCCCGTCTTCTGTTTAGATAACTTCGTCTCTTTCCCAACC
151	NDI1-F1(PGK)	GTAATTATCTACTTTTTACAACAAATATAACAAAATGCTATCGAAGAATTTGTATAGTAACAAGAG
152	NDI1-R1(pYX212t)	GGTCGACGCGTAAGCTTGTGGGCCCTACTATAATCCTTTAAAAAAGTCTCTTTTGAAAAATG
153	PRM9t-F1	ACAGAAGACGGGAGACACTAGCAC
154	PRM9t-R1	ATTTTCAACATCGTATTTTCCGAAG
155	Down-YPRCt3-F1(PRM9t)	CTTCGGAAAATACGATGTTGAAAATGGACGTCAGCACTGTACTTGTTTTTGC
156	Down-YPRCt3-R1	GGTATTACTCGAGCCCGTAATACAAC
157	Diagnostic-F1(YPRCτ3)	GATATAGAAACCATTCCCGAAATATC
158	Diagnostic-R1(YPRCτ3)	GGATAAGTAACATCCCGTGAATC
159	PDA1-F1(TPIp)	GCTTAAATCTATAACTACAAAAAACACATACATAAACTAAAAATGCTTGCTGCTTCATTCAAACGCC
160	PDA1-R1(FBA1t)	CAATACTCATTAAAAAACTATATCAATTAATTTGAATTAACTTAATCCCTAGAGGCAAAACCTTGC
161	ACO2-F1(PGK1p)	GAAGTAATTATCTACTTTTTACAACAAATATAACAAAATGCTATCTTCAGCTAATAGGTTTTATATAAAG

162	ACO2-R1(CYC1t)	CATAACTAATTACATGACTCGAGGTCGACGGTATCTTATTCGTTTCTTCGTATATTACCAATATAATTG
163	CAR1-F(TPI)	CTACAAAAAACACATACATAAACTAAAAATGGAAACAGGACCTCATTACAACTAC
164	CAR1-R(CYC1t)	GACATAACTAATTACATGACTCGAGGTCGACGGTATCCTACAATAAGGTTTCACCCAATGC
165	MTH1-F1(TEF1p)	GAAAGCATAGCAATCTAATCTAAGTTTTAATTACAAAATGTTTGTTTCACCACCACCAGC
166	MTH1-R1(PRM9t)	GTGCTAGTGTCTCCCGTCTTCTGTTCAGGATACTGAATCCGGCTGCC

Supplementary Table 5: Codon optimized genes used in this study

Genes	Nucleotide sequence (5' to 3')
MLS-argB$_{Cg}$	ATGTTATCCTTGAGACAATCCATCAGATTTTTCAAACCTGCTACCAGAACCTTATGCTCTTCCAGATACTTGTTAATGA<br>ATGACTTAATCAAGGACTTGGGTTCTGAAGTTAGAGCAAATGTCTTAGCTGAAGCATTGCCATGGTTGCAACATTTCA<br>GAGATAAGATCGTTGTCGTAAAGTATGGTGGTAACGCCATGGTCGATGACGATTTGAAAGCTGTTTTTGCTGCAGATG<br>TTGTCTTCTTAAGAACTGTTGGTGCTAAGCCAGTAGTTGTCCACGGTGGTGGTCCTCAAATATCAGAAATGTTGAACAG<br>AGTTGGTTTACAGGGTGAATTCAAAGGTGGTTTCAGAGTAACTACACCTGAAGTTATGGACATCGTCAGAATGGTATT<br>GTTTGGTCAAGTCGGTAGAGATTTGGTTGGTTTAATTAACTCCCATGGTCCATACGCAGTTGGTACTAGTGGTGAAGAT<br>GCCGGTTTATTCACAGCTCAAAAGAGAATGGTTAATATAGACGGTGTCCCTACAGATATCGGTTTAGTAGGTGACATT<br>ATAAACGTTGATGCATCTTCATTGATGGATATCATTGAAGCCGGTAGAATCCCAGTAGTTTCCACCATTGCCCCTGGTG<br>AAGACGGTCAAATCTATAATATCAACGCTGATACTGCCGCTGGTGCATTGGCAGCCGCTATTGGTGCTGAAAGATTGT<br>TGGTATTGACAAACGTTGAAGGTTTATACACCGACTGGCCTGATAAGTCCAGTTTGGTTTCTAAAATTAAGGCAACCG<br>AATTAGAAGCCATATTGCCAGGTTTAGATTCTGGTATGATTCCTAAGATGGAATCATGTTTGAACGCTGTCAGAGGTG<br>GTGTATCTGCAGCCCATGTTATTGACGGTAGAATAGCACACTCAGTTTTGTTAGAATTGTTAACCATGGGTGGTATAGG<br>TACTATGGTTTTACCTGATGTCTTCGACAGAGAAAACTATCCAGAAGGTACAGTATTCAGAAAAGACGACAAGGACGG<br>TGAATTATAA
MLS-argJ$_{Cg}$	ATGTTGTCCTTGAGACAATCCATCAGATTCTTCAAACCTGCCACCAGAACTTTATGCTCCTCCAGATACTTGTTAATGG<br>CCGAAAAAGGTATAACAGCTCCAAAAGGTTTCGTTGCCTCTGCTACTACAGCCGGTATCAAGGCTTCAGGTAATCCAG<br>ATATGGCATTGGTTGTCAACCAAGGTCCTGAATTTTCTGCTGCAGCCGTTTTCACTAGAAATAGAGTCTTTGCTGCACC<br>TGTTAAAGTCTCTAGAGAAAACGTTGCTGATGGTCAAATTAGAGCTGTCTTGTATAATGCTGGTAATGCAAACGCCTGT<br>AACGGTTTACAAGGTGAAAAGGATGCAAGAGAATCCGTAAGTCATTTGGCCCAAAATTTGGGTTTAGAAGATTCCGAC<br>ATCGGTGTTTGCAGTACAGGTTTGATTGGTGAATTGTTGCCAATGGATAAGTTGAACGCTGGTATCGACCAATTGACCG<br>CCGAAGGTGCTTTAGGTGACAACGGTGCCGCTGCAGCCAAAGCTATCATGACCACTGATACCGTTGACAAGGAAACTG<br>TAGTTTTTGCAGATGGTTGGACAGTAGGTGGTATGGGTAAAGGTGTTGGTATGATGGCACCTTCATTGGCCACCATGTT<br>AGTATGTTTAACAACCGATGCCTCCGTTACTCAAGAAATGGCTCAAATTGCTTTGGCAAATGCCACCGCTGTCACTTTC<br>GACACATTAGATATAGACGGTTCTACATCAACCAACGATACTGTTTTCTTGTTAGCATCTGGTGCCTCAGGTATCACTC<br>CAACACAAGATGAATTGAATGACGCTGTTTACGCTGCATGCTCTGATATTGCCGCTAAATTACAAGCAGACGCCGAAG<br>GTGTTACAAAGAGAGTAGCAGTTACCGTCGTAGGTACTACAAATAACGAACAAGCTATTAATGCAGCCAGAACAGTT<br>GCAAGAGATAACTTGTTTAAATGTGCCATGTTCGGTTCTGACCCAAATTGGGGTAGAGTCTTAGCTGCAGTTGGTATGG<br>CTGATGCAGACATGGAACCTGAAAAGATATCCGTCTTTTTCAACGGTCAAGCTGTATGCTTGGATAGTACTGGTGCTCC<br>TGGTGCAAGAGAAGTCGACTTGTCTGGTGCTGATATTGACGTTAGAATAGATTTGGGTACTTCAGGTGAAGGTCAAGC<br>AACAGTTAGAACCACTGATTTGTCCTTTAGTTACGTCGAAATTAATTCCGCTTACTCTTCATAA
argC$_{Cg}$	ATGATAATGCACAATGTCTATGGTGTTACAATGACTATTAAGGTCGCAATCGCAGGTGCCTCAGGTTACGCAGGTGGT<br>GAAATCTTGAGATTGTTATTGGGTCATCCAGCATATGCCTCTGGTGAATTAGAAATAGGTGCATTGACCGCTGCATCCA

	CTGCCGGTAGTACATTGGGTGAATTGATGCCACATATTCCTCAATTAGCTGATAGAGTTATACAAGACACTACAGCTG<br>AAACATTGGCAGGTCATGATGTTGTCTTTTTAGGTTTGCCACACGGTTTCTCAGCAGAAATAGCCTTACAATTGGGTCC<br>TGATGTCACAGTAATCGATTGTGCCGCTGACTTTAGATTACAAAATGCAGCCGACTGGGAAAAATTCTATGGTTCCGA<br>ACATCAAGGTACCTGGCCATACGGTATTCCAGAAATGCCTGGTCACAGAGAAGCCTTGAGAGGTGCTAAGAGAGTTGC<br>AGTCCCAGGTTGCTTTCCTACAGGTGCTACCTTAGCATTATTGCCAGCCGTTCAAGCTGGTTTGATCGAACCTGATGTA<br>TCTGTAGTTTCAATTACCGGTGTTTCCGGTGCAGGTAAAAAGGCTAGTGTTGCCTTATTGGGTTCTGAAACTATGGGTT<br>CATTGAAGGCATACAACACCTCAGGTAAACATAGACACACTCCAGAAATCGCTCAAAACTTGGGTGAAGTTTCTGACA<br>AACCAGTAAAGGTTTCATTCACACCTGTTTTAGCTCCATTGCCTAGAGGTATTTTAACCACTGCTACAGCACCTTTGAA<br>AGAAGGTGTCACCGCCGAACAAGCCAGAGCTGTTTACGAAGAATTCTACGCTCAAGAAACTTTCGTCCATGTATTACC<br>AGAAGGTGCCCAACCTCAAACACAAGCTGTTTTGGGTTCCAACATGTGTCACGTTCAAGTCGAAATTGATGAAGAAGC<br>TGGTAAAGTATTGGTTACTAGTGCAATCGACAATTTGACTAAGGGTACAGCAGGTGCTGCAGTTCAATGCATGAACTT<br>ATCTGTCGGTTTTGATGAAGCCGCTGGTTTGCCACAAGTCGGTGTAGCTCCTTAA
argD$_{Cg}$	ATGTCTACATTGGAAACCTGGCCTCAAGTCATCATCAACACATACGGTACTCCTCCTGTCGAATTGGTCTCTGGTAAAG<br>GTGCTACAGTAACCGATGACCAGGGTAACGTTTACATCGATTTGTTGGCTGGTATAGCAGTTAACGCCTTGGGTCATGC<br>TCACCCAGCAATAATCGAAGCTGTAACTAACCAAATAGGTCAATTGGGTCATGTTTCTAACTTATTTGCATCAAGACCT<br>GTTGTCGAAGTTGCCGAAGAATTAATTAAGAGATTCTCTTTGGATGACGCAACATTAGCTGCACAAACCAGAGTTTTCT<br>TTTGTAATTCAGGTGCAGAAGCCAACGAAGCCGCTTTTAAAATCGCTAGATTGACAGGTAGATCCAGAATTTTAGCAG<br>CCGTTCATGGTTTCCACGGTAGAACCATGGGTAGTTTGGCATTAACTGGTCAACCAGATAAGAGAGAAGCATTTTTGC<br>CAATGCCTTCCGGTGTTGAATTCTATCCTTACGGTGACACTGACTATTTGAGAAAAATGGTCGAAACCAATCCAACTGA<br>TGTAGCTGCAATCTTTTTAGAACCTATTCAAGGTGAAACAGGTGTAGTTCCAGCCCCTGAAGGTTTCTTGAAGGCTGTT<br>AGAGAATTGTGTGATGAATACGGTATCTTGATGATCACTGACGAAGTACAAACAGGTGTTGGTAGAACCGGTGACTTT<br>TTCGCACATCAACACGATGGTGTCGTACCAGACGTTGTCACTATGGCTAAAGGTTTGGGTGGTGGTTTACCTATTGGTG<br>CCTGCTTGGCTACAGGTAGAGCCGCTGAATTAATGACCCCAGGTAAACATGGTACTACATTTGGTGGTAACCCTGTTG<br>CTTGTGCAGCCGCTAAAGCAGTCTTGTCAGTAGTTGATGACGCATTTTGCGCCGAAGTTGCTAGAAAGGGTGAATTATT<br>CAAGGAATTGTTGGCTAAGGTTGATGGTGTCGTAGACGTCAGAGGTAGAGGTTTGATGTTAGGTGTTGTCTTGGAAAG<br>AGATGTCGCAAAGCAAGCCGTATTGGACGGTTTTAAACACGGTGTTATTTTAAATGCTCCAGCAGATAACATCATTAG<br>ATTGACTCCACCTTTAGTCATAACAGATGAAGAAATTGCCGACGCTGTTAAAGCAATTGCCGAAACAATAGCTTAA
HaAOX1	ATGTATCCTACTTCTGGTTGTGCTAGAGTTTTGATGGCTTGTCCTGCTCCTGCTATGTTGAGAGGTCCTTTGTTGAGACC<br>TTCCACTACTGCTATTAGAGGTTTGAGAGGTTCCCCATTGTTATATCATTACGCTGCAACTTCCAATAGTAACATGAGA<br>TACTTTTCTTCAACATCCAGAAGATGGATCAAAGAATTTTTCGCACCACCTAAGGAAACAGATCATATAGTAGAAAGT<br>GTTACTACATGGAAACACCCTGTTTTCACCGAAAAACAAATGAAGGAAATTGCTATAGCACATAGAGAAGCTAAGAA<br>TTGGTCTGATTGGGTTGCATTAGGTACAGTCAGATTTTTGAGATGGGCTACAGACTTAGCAACCGGTTACAGACACGC<br>CGCTCCAGGTAAACAAGGTGTTGAAGTCCCTGAACAATTCCAAATGACCGAAAGAAAGTGGGTTATCAGATTCATTTT<br>CTTGGAAACTGTCGCTGGTGTACCAGGTATGGTTGGTGGTATGTTGAGACATTTGAGATCTTTGAGAAGAATGAAGAG<br>AGATAACGGTTGGATTGAAACCTTGTTAGAAGAAGCATATAACGAAAGAATGCACTTGTTATCATTTTTGAAATTGGC<br>CCAACCAGGTTGGTTCATGAGATTAATGGTATTGGGTGCTCAAGGTGTTTTCTTTAACGGTTTCTTTATCTCTTACTTGA<br>TCTCACCTAGAACATGTCATAGATTTGTCGGTTATTTGGAAGAAGAAGCAGTAATGACCTACACTCACGCCATAAAAG

	ATTTGGAATCTGGTAAATTGCCAAATTGGGCCAACCAACCAGCTCCTGACATTGCCGTTGCTTATTGGCAAATGCCTGA AGGTAAAAGAACTATATTGGATTTGTTGTACTACATAAGAGCAGACGAAGCCAAGCATAGAGAAGTTAATCACACATT AGCAAACTTGAAACAAGGTGTCGATCCAAATCCTTATGCAGCCAAGTACGACAACCCAGAAGCCCCTCATCCTACTAA GTCAGCAGAAATTGTCAAGCCTACAGGTTGGGAAAGAGACGAAGTTATCTAA

Supplementary Table 6: Physiological characterization of strains harboring *PDA1* and *PDA1 [S313A]*

Strains	$\mu_{max}(h^{-1})$	r_{ORN} [a]	r_{EOH} [b]	r_{glu} [b]	$Y_{X/S}$ [c]	$Y_{ORN/S}$ [d]	$Y_{EOH/S}$ [c]
M1cM2q	0.38 ± 0.01	33.5 ± 10.0	1.1 ± 0.0	2.8 ± 0.1	0.1 4 ± 0.01	11.88 ± 3.76	0.38 ± 0.01
M1cM2qM3a	0.36 ± 0.02	66.9 ± 7.9	1.2 ± 0.1	3.0 ± 0.5	0.12 ± 0.02	22.34 ± 2.56	0.39 ± 0.08
M1cM2qM3b	0.37 ± 0.01	57.6 ± 7.5	1.2 ± 0.1	2.9 ± 0.1	0.13 ± 0.01	19.82 ± 3.94	0.40 ± 0.03

[a]The uptake and generation rates are given in mg/g dry cell weight per h;
[b]The uptake and generation rates are given in g/g dry cell weight per hour;
[c]Yield from glucose, g/g;
[d]Yield from glucose, mg/g;

Supplementary Table 7: Physiological characterization of strains harboring *MTH1-ΔT*, *HaAOX1* and the *KGD2* deletion

Strains	$\mu_{max}(h^{-1})$	r_{ORN} [a]	r_{EOH} [b]	r_{glu} [b]	$Y_{X/S}$ [c]	$Y_{ORN/S}$ [d]	$Y_{EOH/S}$ [c]
M1dM2q	0.32 ± 0.08	50.7 ± 6.6	1.3 ± 0.3	3.3 ± 0.6	0.09 ± 0.01	15.25 ± 0.86	0.40 ± 0.03
M1dM2qM3c	0.27± 0.07	59.3 ± 3.0	0.7 ± 0.0	2.6 ± 0.3	0.17 ± 0.04	22.98 ± 0.00	0.29 ± 0.15

M1dM2qM3e	0.10 ± 0.00	13.8 ± 2.1	ND[e]	0.2 ± 0.0	0.49 ± 0.05	67.32 ± 0.76	ND
M1dM2qM3f	0.31 ± 0.03	11.2 ± 4.1	0.9 ± 0.2	2.2 ± 0.0	0.14 ± 0.01	5.03 ± 1.81	0.41 ± 0.09

[a]The uptake and generation rates are given in mg/g dry cell weight per h;
[b]The uptake and generation rates are given in g/g dry cell weight per hour;
[c]Yield from glucose, g/g;
[d]Yield from glucose, mg/g;
[e]ND: not detected.

Supplementary Table 8: L-ornithine production with all engineered strains

Strains	DCW g/L[a]		L-ornithine titre mg/L		Yield mg/g DCW	
	Mean.[b]	S.D[c]	Mean.	S.D	Mean.	S.D
Control	4.4	0.1	10.1	0.6	2.3	0.1
M1a	4.5	0.0	23.9	2.4	5.3	0.6
M1b	4.2	0.1	42.0	0.5	9.9	0.2
M1c	4.4	0.2	45.2	3.4	10.3	0.2
M1cM2a	4.4	0.1	41.8	2.3	9.6	0.7
M1cM2b	4.3	0.1	39.0	1.1	9.0	0.4
M1cM2c	4.8	0.0	42.1	3.4	8.8	0.8
M1cM2d	3.7	0.2	37.2	0.8	10.2	10.2
M1cM2e	4.6	0.4	45.0	6.2	9.9	1.8
M1cM2f	3.9	0.2	59.0	2.6	15.0	1.5
M1cM2g	3.8	0.0	80.2	4.7	21.2	1.5
M1cM2h	4.2	0.6	115.9	1.1	27.9	4.2
M1cM2i	4.2	0.7	80.7	11.5	19.5	1.3
M1cM2j	1.6	0.0	20.2	1.4	12.4	1.2

M1cM2k	4.8	0.1	148.9	6.0	30.8	1.6
M1cM2l	4.3	0.6	173.1	7.3	41.3	2.4
M1cM2m	4.4	0.2	142.4	6.7	32.2	1.9
M1cM2n	4.4	0.5	147.5	9.9	33.7	5.0
M1cM2o	4.3	0.2	116.9	11.3	27.0	3.0
M1cM2p	4.1	0.4	161.8	19.6	39.0	2.6
M1cM2q	4.5	0.0	192.0	15.4	42.8	3.5
M1cM2qM3a	4.2	0.1	245.2	15.4	58.4	1.9
M1cM2qM3b	4.4	0.6	264.4	11.7	61.0	7.1
M1cM2qM3c	4.4	0.6	258.1	13.2	59.4	4.9
M1cM2qM3d	4.0	0.7	278.2	14.2	70.3	13.7
M1cM2qM3e[d]	8.9	0.5	778.2	45.4	87.2	0.4
M1dM2qM3e[d]	8.8	0.8	1041.3	46.6	118.1	5.7
M1cM2qM3f	2.7	0.1	130.7	8.8	47.6	2.6

[a] The dry cell weight was calculated according to the following formula: DCW (g/L) = OD_{600}* 0.7 as a linear relationship of DCW (g/L) = OD_{600}* (0.65 to 0.8) was observed in our experiments; [b] Mean of at least three biological replicates; [c] standard derivation; [d] cultures were sampled after 108 h of growth as *MTH1*-Δ*T* over-expression in strain M1cM2qM3e and M1dM2qM3e impaired the glucose uptake.

Supplementary Methods

Plasmid construction

All the plasmids used in this study can be found in Supplementary Table 3. Plasmids (GO1, GO2, GO3, GO4, YO1, YO2, YO3 YO4 and YO3) were constructed according to the MOPE strategy and DNA assembler[3, 4]. All of the primers used for DNA manipulation are listed in Supplementary Table 4. The gene expressing modules consisted of a promoter, a structural gene, a terminator, and the promoter of the next module for homologous recombination. The promoters P_{TEF1}, P_{TDH3}, $P_{PGK1}$and P_{HXT7}, terminators T_{FBA1}, T_{CYC1} ,T_{TDH2} and T_{ADH2}, were PCR-amplified from the genomic DNA of *S. cerevisiae* CEN.PK.113-5D according to the sequence information from[5]. The *TPIp* and the terminator *pYX212t* were PCR-amplified from plasmid pYX212. Genes *ARG2, ARG5,6, ARG7, ARG8, CIT1, ACO2, IDP1, PYC2, GLT1* and *GLN1* were amplified from the genomic DNA of *S. cerevisiae* CEN.PK.113-5D. *PDA1* and mutated *mPDA1* were PCR-amplified from plasmid pRS416-PDA1 and pRS416-PDA1 [S313A] respectively[2]. *HaAOX1*, $argJ_{Cg}$, $argC_{Cg}$, $argD_{Cg}$, and $argB_{Cg}$ were codon-optimized and purchased from GenScript (all sequences can be found in Supplementary Table 5). $argA_{Ec}$ and $argB_{Ec}$ were PCR-amplified from the genomic DNA of *E. coli*. The mutated *MTH1-ΔT* was PCR-amplified from the genomic DNA of *S. cerevisiae* TAM[6]. $argB_{Cg}$ and $argJ_{Cg}$ were targeted to the mitochondria using the N-terminal mitochondrial localization signal from subunit IV of the yeast cytochrome c oxidase (CoxIV)[7, 8]. All modules were constructed with the one-step PCR strategy similar to overlap extension PCR. The expression modules were co-transformed by electroporation with linearized vector pYX212 or p423GPD into *S. cerevisiae* BY4741, and the recombinants appeared on the corresponding plates after 2-4 days. Selected colonies formed on the plates were cultured in 5 ml of YPD liquid medium at 30 °C for 72 h. Recovered plasmids were checked by PCR to verify the assembled pathways. Alternatively, positive plasmids were also transformed into *E. coli* DH5α, recovered, digested by restriction endonucleases, and analyzed by gel electrophoresis. Other plasmids used in this study were constructed according to the regular restriction-enzyme based cloning.

Promoter replacement and chromosome integration of target genes

To replace the *ARG3* promoter, the *HXT1* promoter and *KEX2* promoter were amplified from genomic DNA by PCR. The DNA cassette including the new promoter, the *kanMX* cassette and parts up- and downstream of the ARG3 promoter was constructed following the strategy of MOPE. Following the transformation of these cassettes into *S. cerevisiae*, the correct transformants were selected and verified by colony PCR. Following a similar strategy, *ORT1*,

ODC1, *AGC1*, *GDH1*, *GDH2*, *GDH3*, *HaAOX1* and *MTH1*-Δ*T* were integrated into the chromosome of *S. cerevisiae* background strains, yielding strains with modification in the *URA3*, *YPRCτ3* or *KGD2* sites of the chromosome (Supplementary Table 1)[9]. Similar to $argB_{Cg}$ and $argJ_{Cg}$, *GDH1* and *GDH2* were targeted to the mitochondria using the N-terminal mitochondrial localization signal from subunit IV of the yeast cytochrome c oxidase (CoxIV). Variation combinations of plasmids and background strains yielded L-ornithine producing strains (Supplementary Table 2).

Cell growth media

Yeast strains without plasmids were maintained on YPD plates containing 10 g l^{-1} yeast extract, 20 g l^{-1} casein peptone, 20 g l^{-1} glucose and 20 g l^{-1} agar. Plasmid carrying yeast strains were selected on synthetic dextrose (SD) agar containing 6.9 g l^{-1} yeast nitrogen base w/o amino acids (Formedium, Hunstanton, UK), 20 g l^{-1} glucose, and 20 g l^{-1} agar. Strains containing the *kanMX* cassette were selected on YPD plates containing 200 mg l^{-1} G418 (Formedium, Hunstanton, UK). Defined minimal medium (Delft medium) as described before was used for both batch cultivations and fed-batch fermentations of L-ornithine producing strains[10]. Luria Bertani (LB) broth with 80 mg l^{-1} ampicillin was used for maintenance of *E. coli* DH5α harboring appropriate plasmids.

Shake flask cultivation for L-ornithine production

Shake flask cultivation was used to evaluate the L-ornithine producing strains. 20 ml cultures were started in 100 ml unbaffled cotton-stopped flasks by inoculating an amount of pre-culture that resulted in a final optical density of 0.05 at 600 nm (OD600). The strains were grown at 30 °C with 200 rpm. orbital shaking in defined minimal medium (Delft medium) with 20 g l^{-1} glucose[10]. Samples were taken periodically to measure the cell mass, L-ornithine titre, residual glucose and other metabolites.

Fed-batch cultivation of L-ornithine producing strains

For fed-batch cultivations[10], strains were first grown in a batch culture with Delft medium and then an initial volume of 900 ml Delft media in a 3 l bioreactor was inoculated to a cell density of 0.05. Cells were cultivated at 30 ºC, 600 rpm agitation, 1 vvm air flow, dissolved oxygen above 30% (controlled by adjusting the air flow and agitation) and pH 5.5 (controlled by 2 M KOH). After the glucose and part of the ethanol were consumed, the exponential feed was started.

The temperature, agitation, gassing, pH and composition of the off-gas were monitored and controlled using the DasGip monitoring and control system. The effluent gas from the fermentation was analyzed for real-time determination of oxygen and CO_2 concentration by

DasGip fed-batch pro® gas analysis systems with the off gas analyzer GA4 based on zirconium dioxide and two-beam infrared sensor.

A feed strategy was designed keeping the volumetric growth rate constant. An exponential feed rate ν(*t*) (l h^{-1}) was calculated according to:

$$\nu\ (t)=\frac{Y_{xs}\ \mu_0}{s_f - s_0}\ x_0 V_0 \exp\ (\mu_0 t)$$

where x_0, s_0 and V_0 were the biomass density (g DCW l^{-1}), the substrate concentration (g l^{-1}) and the reactor volume (l) at the start of the fed-batch process, *Yxs* was the respiratory yield coefficient (g glucose $gDCW^{-1}$); s_f was the concentration of the growth limiting substrate (g glucose l^{-1}) in the reservoir; μ_0 the was the specific growth rate (h^{-1}) during the feed phase and *t* the feeding time.

Supplementary References

1. Johansson, N., Persson, K.O., Quehl, P., Norbeck, J. & Larsson, C. Ethylene production in relation to nitrogen metabolism in *Saccharomyces cerevisiae*. *FEMS Yeast Res.* **14**, 1110-1118 (2014).
2. Oliveira, A.P. et al. Regulation of yeast central metabolism by enzyme phosphorylation. *Mol. Syst. Biol.* **8**, 623-623 (2012).
3. Shao, Z., Zhao, H. & Zhao, H. DNA assembler, an in vivo genetic method for rapid construction of biochemical pathways. *Nucleic Acids Res.* **37**, e16 (2009).
4. Zhou, Y.J.J. et al. Modular pathway engineering of diterpenoid synthases and the mevalonic acid pathway for miltiradiene production. *J. Am. Chem. Soc.* **134**, 3234-3241 (2012).
5. Sun, J. et al. Cloning and characterization of a panel of constitutive promoters for applications in pathway engineering in *Saccharomyces cerevisiae*. *Biotechnol. Bioeng.* **109**, 2082-2092 (2012).
6. Oud, B. et al. An internal deletion in *MTH1* enables growth on glucose of pyruvate-decarboxylase negative, non-fermentative *Saccharomyces cerevisiae*. *Micro. Cell. Fact.* **11**, 131 (2012).
7. Avalos, J.L., Fink, G.R. & Stephanopoulos, G. Compartmentalization of metabolic pathways in yeast mitochondria improves the production of branched-chain alcohols. *Nat. Biotechnol.* **31**, 335-341 (2013).
8. Maarse, A.C. et al. Subunit IV of yeast cytochrome c oxidase: cloning and nucleotide sequencing of the gene and partial amino acid sequencing of the mature protein. *EMBO J.* **3**, 2831-2837 (1984).
9. Bai Flagfeldt, D., Siewers, V., Huang, L. & Nielsen, J. Characterization of chromosomal integration sites for heterologous gene expression in *Saccharomyces cerevisiae*. *Yeast* **26**, 545-551 (2009).
10. Scalcinati, G. et al. Dynamic control of gene expression in Saccharomyces cerevisiae engineered for the production of plant sesquitepene α-santalene in a fed-batch mode. *Metab. Eng.* **14**, 91-103 (2012).
